# The Long Non-coding RNA NRIR Drives IFN-Response in Monocytes: Implication for Systemic Sclerosis

**DOI:** 10.3389/fimmu.2019.00100

**Published:** 2019-01-31

**Authors:** Barbara Mariotti, Nila Hendrika Servaas, Marzia Rossato, Nicola Tamassia, Marco A. Cassatella, Marta Cossu, Lorenzo Beretta, Maarten van der Kroef, Timothy R. D. J. Radstake, Flavia Bazzoni

**Affiliations:** ^1^General Pathology Section, Department of Medicine, University of Verona, Verona, Italy; ^2^Department of Rheumatology and Clinical Immunology, University Medical Center Utrecht, Utrecht, Netherlands; ^3^Laboratory of Translational Immunology, University Medical Center Utrecht, Utrecht, Netherlands; ^4^Department of Biotechnology, University of Verona, Verona, Italy; ^5^Scleroderma Unit, Fondazione IRCCS Ca' Granda Ospedale Maggiore Policlinico di Milano, Referral Center for Systemic Autoimmune Diseases, Milan, Italy

**Keywords:** long non-coding RNAs, monocytes, systemic sclerosis, interferon, NRIR

## Abstract

TLR4 activation initiates a signaling cascade leading to the production of type I IFNs and of the downstream IFN-stimulated genes (ISGs). Recently, a number of IFN-induced long non-coding RNAs (lncRNAs) that feed-back regulate the IFN response have been identified. Dysregulation of this process, collectively known as the “Interferon (IFN) Response,” represents a common molecular basis in the development of autoimmune and autoinflammatory disorders. Concurrently, alteration of lncRNA profile has been described in several type I IFN-driven autoimmune diseases. In particular, both TLR activation and the upregulation of ISGs in peripheral blood mononuclear cells have been identified as possible contributors to the pathogenesis of systemic sclerosis (SSc), a connective tissue disease characterized by vascular abnormalities, immune activation, and fibrosis. However, hitherto, a potential link between specific lncRNA and the presence of a type I IFN signature remains unclear in SSc. In this study, we identified, by RNA sequencing, a group of lncRNAs related to the IFN and anti-viral response consistently modulated in a type I IFN-dependent manner in human monocytes in response to TLR4 activation by LPS. Remarkably, these lncRNAs were concurrently upregulated in a total of 46 SSc patients in different stages of their disease as compared to 18 healthy controls enrolled in this study. Among these lncRNAs, Negative Regulator of the IFN Response (NRIR) was found significantly upregulated *in vivo* in SSc monocytes, strongly correlating with the IFN score of SSc patients. Weighted Gene Co-expression Network Analysis showed that NRIR-specific modules, identified in the two datasets, were enriched in “type I IFN” and “viral response” biological processes. Protein coding genes common to the two distinct NRIR modules were selected as putative NRIR target genes. Fifteen *in silico*-predicted NRIR target genes were experimentally validated in NRIR-silenced monocytes. Remarkably, induction of CXCL10 and CXCL11, two IFN-related chemokines associated with SSc pathogenesis, was reduced in NRIR-knockdown monocytes, while their plasmatic level was increased in SSc patients. Collectively, our data show that NRIR affects the expression of ISGs and that dysregulation of NRIR in SSc monocytes may account, at least in part, for the type I IFN signature present in SSc patients.

## Introduction

Toll-like receptor 4 (TLR4) is a member of the pattern recognition receptors (PRR) family, which detects conserved structures found in a broad range of pathogens and triggers innate immune responses. TLR4 signals through two major pathways: (i) the MyD88-dependent pathway, that elicits the release of pro-inflammatory cytokines, such as TNF-α, IL-6, and IL-12p40; (ii) the TRIF (Toll/IL-1R domain-containing adaptor-inducing IFN-beta)-dependent pathway, that contributes to pro-inflammatory cytokine responses and, most importantly, induces type I IFN responses, particularly IFN-β (1). IFNs confer their activity by regulating networks of interferon-stimulated genes (ISGs), a process that requires *de novo* transcription and translation of both IFN and downstream ISGs (2). Other than being activated by different exogenous pathogen-associated molecular patterns (PAMPs), the IFN pathway is activated also by TLR4 ligation of endogenous danger-associated molecular patters (DAMPs) released upon cell damage or stress (3, 4). Thus, TLR4-mediated activation of innate immunity plays a key role not only in host defense against pathogens but also in numerous autoimmune diseases, including systemic sclerosis (SSc) (5). Indeed, endogenous ligand-induced TLR4 activation has been recognized as a key player driving the persistent fibrotic response in SSc (5–7). Different endogenous TLR4 ligands, including fibronectin extra domain A (FnEDA) and S100A8/A9, are indeed increased in the circulation of SSc patients and have been correlated with fibrotic-related clinical complications (8, 9). Moreover, activation of TLR4 response leads to transforming growth factor-β production, a crucial mediator for fibrosis development in SSc (10).

Likewise, production of type I interferon is closely linked to TLR4-mediated innate immune signaling in SSc (11–13). In fact, several lines of evidence suggest that both the IFN network and monocytes are implicated in SSc immune-pathogenesis. First, the development of SSc has been reported in patients undergoing IFN treatment (14) and IFN-α injections worsen SSc-related clinical features (15). Most importantly, increased expression of type I IFN-regulated genes, known as “type I IFN signature,” is a hallmark of SSc, and type I IFN signature is present both in the fibrotic skin and in peripheral blood cells (11, 13), as well as in monocytes of SSc patients from the earliest phases of the disease, even before the skin fibrosis is evident (16). Moreover, in the fibrotic subsets of SSc patients we identified an increase in non-classical monocytes spontaneously producing the IFN-responsive CXCL10 (17), a chemokine associated with faster progression rate from pre-fibrotic SSc to worse disease stages (18).

The IFN pathway downstream TLR4 activation has been focus of intense investigation and a number of known protein-mediated mechanisms that mediate the complex signaling pathways and gene expression programs involved in the interferon response have been identified (2). Recent studies point at long non-coding RNAs (lncRNAs) as a novel class of IFN pathway regulatory molecules (19). LncRNAs are RNA transcripts longer than 200 nucleotides, characterized by lacking protein coding capability, but able to regulate gene expression both at the transcriptional and post-transcriptional levels (20). Existing data indicate that lncRNAs are critically involved in various biological and immunological processes (21), including several pathways related to innate immunity (22–29). However, with respect to the IFN response, while IFN-induced changes in the expression of protein-coding RNAs and their functional outcome have been well-documented, our knowledge of the impact of IFNs on lncRNA genes is highly incomplete. Moreover, the involvement of lncRNAs in diseases such as SSc, where both TLR4 and type I IFN concur to disease pathogenesis, is still unexplored.

This study aims to investigate the profile and the role of lncRNAs in the IFN response initiated by TLR4 activation of primary human monocytes and their implication in the immune dysregulation present in SSc patients.

## Materials and Methods

### Patients

Patients affected by systemic sclerosis (SSc) and sex- and age-matched healthy controls (HC) were obtained from the University Medical Center Utrecht (UMCU), The Netherlands, and the Scleroderma Unit of Fondazione IRCCS Policlinico of Milan, Italy. Patients fulfilling the ACR/EULAR 2013 criteria (30) were classified in relation to the extent of skin fibrosis as limited cutaneous (lcSSc) or diffuse cutaneous SSc (dcSSc) (31); patients satisfying the classification criteria without skin fibrosis were referred to as non-cutaneous SSc (ncSSc). Additionally, early SSc (eaSSc) subjects were defined as patients presenting with Raynaud's phenomenon and SSc-specific autoantibodies and/or typical nailfold videocapillaroscopy abnormalities (32). Three separate cohorts, herein named “definite SSc” cohort, “non-fibrotic SSc” cohort, “SSc cohort 3,” were recruited for the current study. Demographics and clinical characteristics of the three cohorts are reported in Tables 1–3. All patients and healthy donors signed an informed consent form approved by the local institutional review boards prior to participation in the study. Samples and clinical information were made de-identified immediately after collection.

**Table 1:** Demographics and clinical characteristics of the donors included in the definite SSc cohort.

**Patient group (*n*)**	**HC (9)**	**ncSSC (7)**	**lcSSc (11)**	**dcSSc (7)**
Age (yr.)	52 (30–64)	45 (26–63)	59 (45–70)	58 (34–72)
Female (*n*, %)	5 (56%)	6 (86%)	8 (73%)	3 (43%)
ANA (*n* pos, %)	–	6 (86%)	10 (91%)	7 (100%)
ACA (*n* pos, %)	–	3 (43%)	6^*^ (55%)	1 (14%)
Scl70 (*n* pos, %)	–	2 (29%)	2^*^ (18%)	4 (57%)
mRSS	–	0	6 (0–12)	14^*^ (5–36)
ILD	–	1 (14%)	2 (18%)	5 (71%)
Disease Duration (yr.)		4 (1–12)	9 (1–19)	10 (2–27)

*Values reported indicate the number (n) of patients and the median for each parameter [Interquartile Range (IQR)], if not otherwise indicated. ACA, anticentromere antibodies; ANA, antinuclear antibodies; dcSSc, diffuse cutaneous SSc; HC, healthy controls; ILD, Interstitial Lung disease; lcSSc, limited cutaneous SSc; mRSS, modified Rodman Skin score; ncSSc, non-cutaneous SSc; pos, positivity; Scl70, anti-topoisomerase antibodies; Yr., years*.

* *1 patient unknown*.

**Table 2:** Demographics and clinical characteristics of the donors included in the non-fibrotic SSc cohort.

**Patient group (*n*)**	**HC (9)**	**eaSSC (11)**	**ncSSc (10)**
Age (yr.)	38 (28–49)	57 (40–77)	52 (25–70)
Female (*n*, %)	9 (100%)	11 (100%)	10 (100%)
ANA (*n* pos, %)	–	10 (91%)	10 (100%)
ACA (*n* pos, %)	–	7 (64%)	8 (80%)
Scl70 (*n* pos, %)	–	2 (18%)	1 (10%)
mRSS	–	0	0
ILD	–	0	0
Disease Duration (yr.)	–	–	Unknown

*Values reported indicate the number (n) of patients and the median for each parameter [Interquartile Range (IQR)], if not otherwise indicated. ACA, anticentromere antibodies; ANA, antinuclear antibodies; eaSSc, early SSc; HC, healthy controls; ILD, Interstitial Lung disease; mRSS, modified Rodman Skin score; ncSSc, non-cutaneous SSc; pos, positivity; Scl70, anti-topoisomerase antibodies; Yr., years*.

**Table 3:** Demographics and clinical characteristics of the donors included in the SSc cohort 3.

**Patient group (n)**	**HC (21)**	**eaSSc (15)**	**ncSSc (27)**	**lcSSc (23)**	**dcSSc (19)**
Age (yr.)	52 (35–82)	62 (25–81)	59 (29–80)	60 (41–80)	52 (27–80)
Female (*n*, %)	19 (90%)	15 (100%)	27 (100%)	22 (96%)	15 (79%)
ANA (*n* pos, %)	–	15 (100%)	26 (96%)	22 (96%)	16 (84%)
ACA (*n* pos, %)	–	12 (80%)	20 (74%)	12 (52%)	0 (0%)
Scl70 (*n* pos, %)	–	2 (13%)	1 (4%)	9 (39%)	11 (58%)
mRSS	–	0	0	4 (0–8)	12 (2–29)
ILD	–	0	2 (7%)	7 (30%)	14 (74%)
Disease Duration (yr.)	–	N.A.	10^*^ (0–29)	16^**^ (1–38)	10 (1–25)

*Values reported indicate the number (n) of patients and the median for each parameter [Interquartile Range (IQR)], if not otherwise indicated. ACA, anticentromere antibodies; ANA, antinuclear antibodies; dcSSc, diffuse cutaneous SSc; eaSSc, early SSc; HC, healthy controls; ILD, Interstitial Lung disease; lcSSc, limited cutaneous SSc; mRSS, modified Rodman Skin score; N.A., not assessed; ncSSc, non-cutaneous SSc; pos, positivity; Scl70, anti-topoisomerase antibodies; Yr., years*.

* *2 patients unknown*.

** *3 patients unknown*.

### Cell Purification and Culture

Human CD14+ monocytes and neutrophils (PMNs) were purified from heparinised whole blood of SSc patients and matched HC or from buffy coats of healthy donors after centrifugation over Ficoll-Paque gradient. Briefly, CD14+ monocytes were purified from PBMCs using the anti-CD14 microbeads (Miltenyi Biotec), on the autoMACs Pro Separator (Miltenyi Biotec) according to manufacturer's protocol. Purity of monocyte preparations was usually >98%. PMNs were recovered after dextran sedimentation and hypotonic lysis of erythrocytes followed by EasySep neutrophil enrichment kit (StemCell Technologies, Vancouver, Canada) (33). Purity of neutrophils preparations was usually 99.7 ± 0.2%.

Monocytes (3 × 10^6^ cells/ml) and PMNs (5 × 10^6^ cells/ml) were cultured in RPMI 1640 (Gibco) supplemented with 10% FCS (<0.5 EU/ml; Sigma-Aldrich) and 2 mM Glu in the presence or absence of 100 ng/ml ultra-pure lipopolysaccharide (LPS, from E. coli strain O111:B4, InvivoGen, San Diego, CA, USA), 5 μM R848 (Invivogen), 1,000 U/ml IFNα CRI003B, Cell Sciences), 100 ng/ml palmitoyl-3-cysteine-serine-lysine-4 (Pam3CSK_4_, Invivogen), 50 μg/ml polynosinic:polycytidylic acids [poly(I:C), Invivogen], as indicated. In selected experiments, CD14+ monocytes were incubated for 30 min with 5 μg/ml Brefeldin A (BFA, Sigma-Aldrich) or 5 μg/ml αIFNAR (PBL InterferonSource, Piscataway, NJ, USA) or its isotype control antibody (mouse IgG2a), before cell stimulation.

### Human Monocyte Transfection

Freshly purified monocytes (8 × 10^6^) were transfected with 200 pmol NRIR-specific Silencer Select siRNA or Silencer Select negative control #2 (both from Ambion, Thermo Scientific), using the Human Monocyte Nucleofector Kit and the AMAXA Nucleofector II device (both from Lonza), according to the manufacturer's protocol. Once transfected, cells were plated in recovery medium [50% RPMI 1640 + 10% FCS + 2 mM Glu, and 50% IMDM (Lonza) + 10% FCS + 2 mM Glu], at 3 × 10^6^ cells/ml overnight. The next day, medium was changed to RPMI 1640 + 10% FCS + 2 mM Glu, and cells were stimulated as indicated. NRIR specific Silencer Select siRNA sequence (34) is reported in Table S1.

### Extraction of Total RNA

Total RNA was purified with the RNeasy Mini Kit (Qiagen), according to the manufacturer's instructions. DNAse treatment (RNAse Free DNase I set, Qiagen) on column was performed. RNA quantification, purity and integrity were assessed at the Nanodrop 2000 spectrophotometer (Thermo Scientific) and by capillary electrophoresis on an Agilent Bioanalyzer (Agilent Technologies), respectively. Purified RNA was used for sequencing analysis or RT-qPCR, as described below.

### RNA Sequencing Analysis

RNA sequencing data of peripheral blood monocytes purified from SSc, together with sex- and age-matched healthy controls (HC) enrolled in the “definite SSc” cohort, were obtained from the University Medical Center Utrecht (UMCU), The Netherlands (35).

RNA sequencing libraries were generated from total RNA extracted from CD14+ monocytes of SSc patients and matched HC enrolled in the “definite SSc” and “non-fibrotic SSc” cohorts, or from RNA pools of three different donors of freshly isolated and LPS-treated monocytes. RNA-seq library preparation was accomplished using the TruSeq RNA Sample Prep Kit v2 (Illumina Inc., San Diego, CA, USA). Libraries were sequenced on a HiSeq 2000 system (Illumina) using pair-end sequencing reads (2 × 90 bp for SSc and matched HC libraries and 2 × 51 bp for resting and LPS-treated monocytes libraries); a minimum of 20 million fragments per sample were analyzed. After quality filtering according to the Illumina pipeline, reads were firstly aligned to the human transcriptome annotated in Ensembl 77 (*Homo sapiens* gene model annotation) and secondly converted to genomic mapping using as reference the human reference genome GrCh38 (Genome Reference Consortium Human build 38) by means of TopHat (v 2.0.14) (36). On average, 23,969,150 (concordant pair alignment rate: 91.84%), 24,404,133 (concordant pair alignment rate: 89.90%), and 43,071,006 (concordant pair alignment rate: 92.67%) paired-reads of the “definite SSc,” “non-fibrotic SSc” and LPS-treated-monocytes dataset, respectively, mapped to the reference genome.

Differential expression analysis was performed using the generalized linear model (GLM) implemented in DESeq2 (v 1.6.3) on the summed exon reads count per gene estimated using HTSeq-count (v 0.6.1p1) (37, 38). Differentially expressed genes were identified from the comparison of each single SSc group and matched HC. Significance was tested using the Wald test. Genes with a log_2_(FC) value ≥0.58 or ≤ -0.58 and a *p* ≤ 0.05, were considered significantly modulated. Differentially expressed genes in LPS-treated monocytes were identified using the Likelihood Ratio Test (LRT). Raw *p*-values from differential expression analyses were adjusted to control the false discovery rate (FDR) using the Benjamini–Hochberg method. Genes with adjusted *p* < 0.05 were considered significantly modulated by LPS. Gene expression levels were expressed as variance stabilized data (vsd) or FPKM, calculated according to DESeq2 instructions. Gene type were associated according to the Ensembl 77 annotation. All genes not belonging to the gene type protein coding and pseudogene and with a transcript length of at least 200 bp were considered as lncRNAs. Raw and processed sequencing data are available from Gene Expression Omnibus under the following accession numbers: GSE123532 and GSE124075.

### Gene Expression Data of PBMC From SLE Patients and Relative Healthy Controls

Gene expression profiles of PBMC purified from systemic lupus erythematosus (SLE) and relative healthy donors (HC) were downloaded from Gene Expression Omnibus Database (GEO number: GSE122459). Gene expression levels and differential expression analysis were retrieved from the dataset present in the GEO database.

### GO-Term and Pathway Enrichment Analysis

Protein coding genes (PCGs) were subjected to Gene Ontology (GO) and pathway enrichment analysis using ToppFun^1^ (39). *p*-value was calculated according to the probability density function and corrected for the False Discovery Rate (FDR) according to Benjamini-Hochberg method. Pathways and GO-terms associated to biological processes (BP) with a FDR≤0.05 were considered significantly enriched.

### Weighted Gene Co-expression Network Analysis

Co-expression networks were generated using WGCNA R-package (40). Signed weighted adjacency matrix of connection strengths was constructed using the soft-threshold approach with a scale-independent topological power β = 18 for LPS-treated and freshly isolated monocytes and β = 13 for the definite SSc data. Genes were aggregated into modules by hierarchical clustering and refined by the dynamic tree cut algorithm. Biological function of each module was evaluated by pathway and BP GO-terms enrichment analysis using ToppFun (39). All terms enriched with a FDR < 0.05, were considered. Redundancy of significantly enriched BP GO-terms was solved by means of REVIGO (41) using the simRel score to assess similarity between two GO-terms (42). NRIR-specific modules were visualized using Cytoscape v3.2.1 (43).

### Gene Expression Analysis by Real-Time PCR

RNA samples were reverse transcribed using 5 ng/μl random primers, 1 U/μl RNase inhibitor (RNAse Out, Invitrogen) and 5 U/μl reverse transcriptase (SuperScript III, Invitrogen), according to manufacturer's instruction. NRIR expression was quantified in duplicates by RT-qPCR from 9 ng RNA-equivalent cDNA in the presence of SYBR Select Master Mix (ThermoFisher Scientific, Applied Biosystems) and 400 nM specific primers (Table S1), on the ViiA™ 7 Real-Time PCR System (ThermoFisher Scientific, Applied Biosystems) using the standard protocol. PCG expression was quantified in duplicates by RT-qPCR from 9 ng RNA-equivalent cDNA in the presence of Fast SYBR Green Master mix (ThermoFisher Scientific, Applied Biosystems) and 200 nM of specific primer pairs (Table S1), on the ViiA™ 7 Real-Time PCR System (ThermoFisher Scientific, Applied Biosystems). Primers were designed using the Oligo Explorer software^2^, for only fifty-six out seventy-nine NRIR putative target genes was possible to design specific primer pairs. Data were analyzed with LinReg PCR 7.0^3^ and Q-Gene software^4^ Gene expression was calculated as mean normalized expression [MNE (44)] units after normalization over the stably expressed RPL32 or ACTIN B.

### Multiplex Immunoassay

CXCL10, CXCL11, and CCL8 concentrations in cell-free supernatants and/or plasma from SSc patients and matched HC enrolled in the “SSc cohort 3” were measured using an in-house developed and validated (ISO9001 certified) multiplex immunoassay (Laboratory of Translational Immunology, University Medical Center Utrecht) based on Luminex technology (xMAP, Luminex Austin TX USA). The assay was performed as previously described (45). Aspecific heterophilic immunoglobulins were pre-absorbed from all plasma samples with heteroblock (Omega Biologicals, Bozeman MT, USA). All samples were measured with the Biorad FlexMAP3D (Biorad laboratories, Hercules USA) in combination with the xPONENT software (v 4.2, Luminex). Data were analyzed by a 5-parametric curve fitting using the Bio-Plex Manager software (v 6.1.1, Biorad).

### Statistical Analysis

Data are expressed as mean ± SEM unless otherwise indicated. Statistical evaluation was determined using the Mann Whitney test or the two-way analysis of variance (ANOVA), followed by Bonferroni post-test, with α set to 0.05. Correlation analysis were performed using the *rcorr()* function in R using the non-parametric Spearman method. Correlation with *p* < 0.05 were considered significant.

## Results

### Identification of LPS-Modulated lncRNAs in Primary Human Monocytes

**Figure 1:**
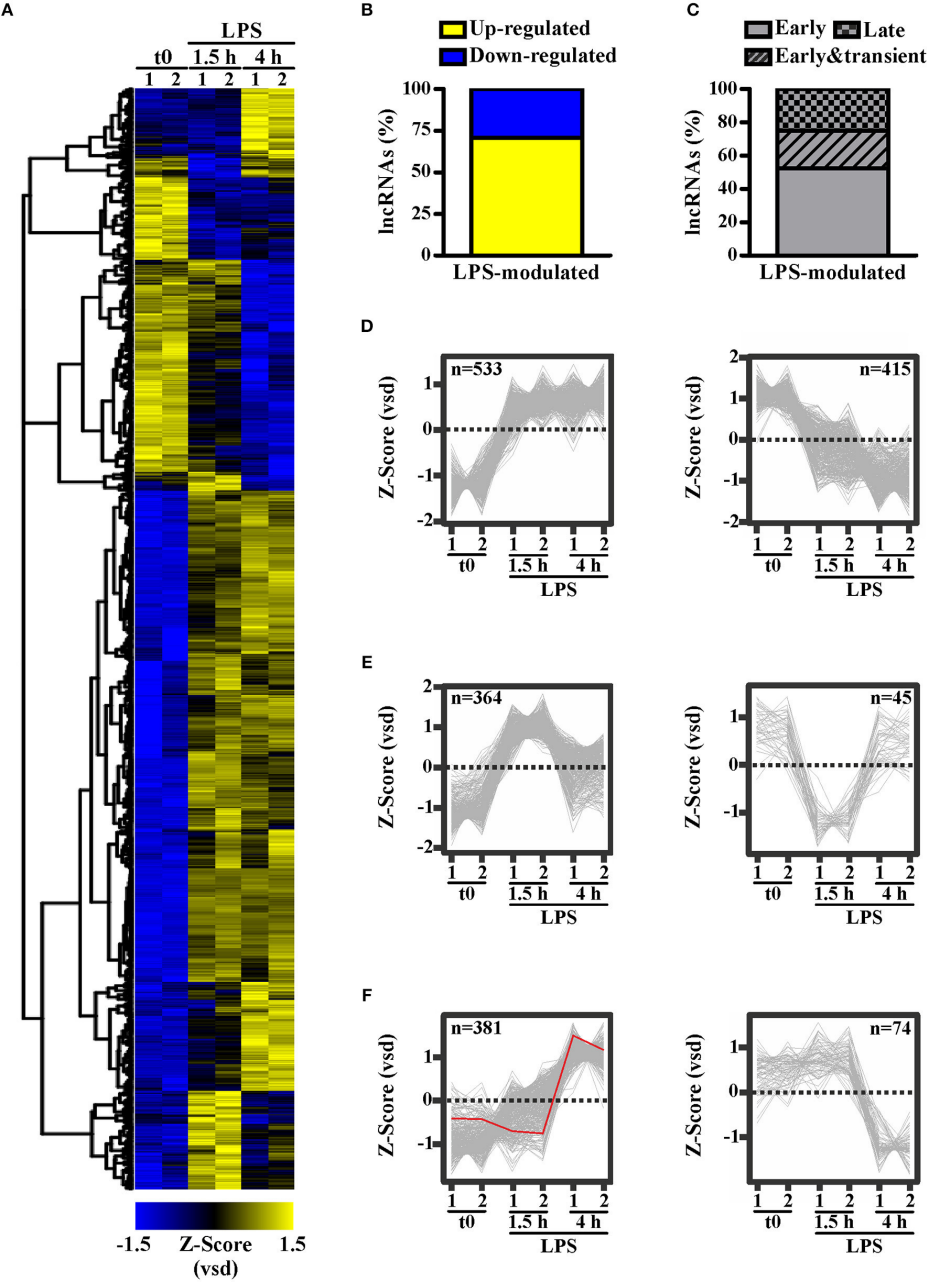
LPS modulates the expression of long noncoding transcripts in human monocytes. CD14+ monocytes were cultured for 1.5 or 4 h with LPS (100 ng/ml) or left untreated (t0). Two pools of three donors for each condition were used to create polyA library for RNA-seq. Sequencing data were analyzed as described in Materials and Methods. The expression levels of the LPS-modulated (adjusted *p* < 0.05) lncRNAs **(A)** are shown as row mean-centered *z*-Score of the variance stabilized data (vsd). **(B)** The percentage of up- and down-regulated lncRNAs modulated by LPS. **(C)** The percentage of early, early & transient and late lncRNAs modulated by LPS. K-means clustering analysis was applied on the significantly modulated lncRNAs. Early modulated **(D)**, early and transiently modulated **(E)** as well as late modulated **(F)** lncRNAs are shown. The expression of each lncRNA belonging to the three groups is shown. LncRNAs up regulated and down regulated by LPS are shown separately. NRIR expression is highlighted in red. LncRNA expression is depicted as row mean-centered *z*-Score of the variance stabilized data (vsd), number of lncRNA belonging to each KMC group is shown.

To identify lncRNAs potentially involved in the responses of peripheral human monocytes downstream TLR4 activation, CD14+ monocytes purified from buffy coats of healthy donors were cultured in the presence or absence of LPS (100 ng/ml) for 1.5 h or 4 h, and subsequently subjected to RNA sequencing. 1,812 transcripts annotated as lncRNAs in Ensemble (Figure 1A) were identified as significantly (p-adj < 0.05) modulated in response to LPS. Specifically, 1278 lncRNAs (i.e., 70.53%) were up-regulated, while 534 lncRNAs (i.e., 29.47%) were down-regulated (Figure 1B). Moreover, K-means clustering arranged the LPS-modulated lncRNAs in three main groups according to their kinetic of expression (Figure 1C): (i) lncRNAs rapidly and consistently modulated by LPS within 1.5 h, representing the majority (52.32%) of LPS-modulated lncRNAs (early group, Figure 1D); (ii) lncRNAs modulated by LPS within 1.5 h in a transient manner (22.57%) (early and transient group, Figure 1E); (iii) lncRNAs modulated by LPS at 4 h (25.11%) (late group, Figure 1F).

### Identification of Type I IFN Signature-Associated lncRNAs

**Figure 2:**
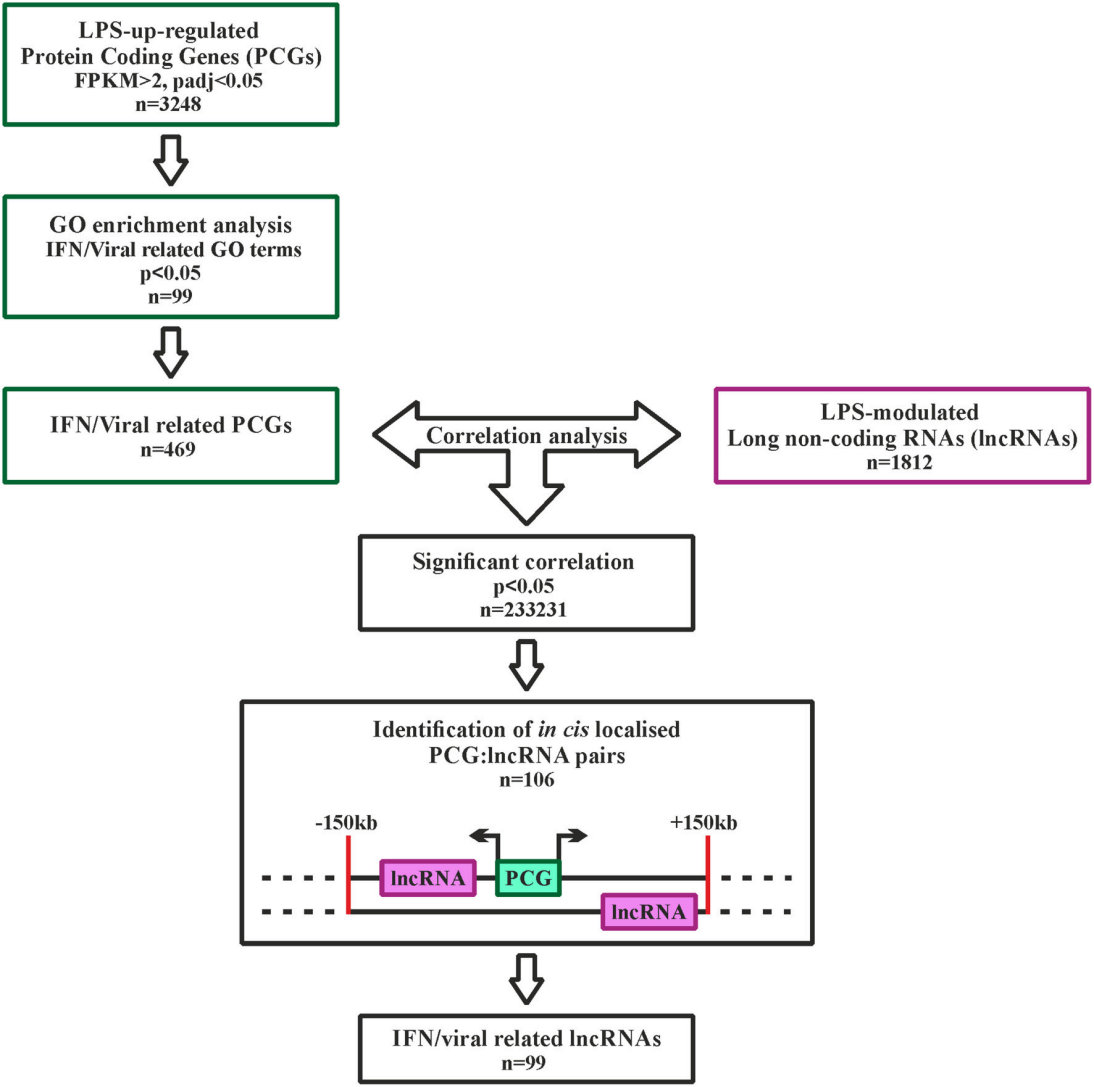
Analysis pipeline to identify IFN/viral-related lncRNAs, modulated by LPS in monocytes. Green squares represent the selection of IFN/viral related protein coding genes, while the purple square represents the selected lncRNAs modulated by LPS. Black squares represent the workflow for integration of protein coding genes and lncRNAs by correlation analysis.

LncRNAs possibly involved in the regulation of type I IFN pathway activated downstream TLR4 were identified using the strategy depicted in Figure 2. Specifically, 3,248 PCGs up-regulated in response to LPS (FPKM > 2) were retrieved and subjected to GO term enrichment analysis. 469 LPS-induced PCGs associated to significantly enriched IFN-response and anti-viral response-related GO-terms were then subjected to correlation analysis with the 1,812 LPS-modulated lncRNAs. Finally, based on the knowledge that lncRNAs can regulate the transcription of PCGs located *in cis* (46), only the lncRNAs localized *in cis* (± 150 Kb) to correlated PCGs were retrieved (*n* = 99) (Figure 2 and Table S2). This group of lncRNAs (*n* = 99) will be referred from now on as the “IFN/viral” lncRNAs.

To verify whether the selected “IFN/viral” lncRNAs were effectively related to the IFN signature in an *in vivo* setting where the IFN pathway is known to play a pathogenetic role, the expression level of the 99 selected lncRNAs was then retrieved and analyzed from the transcriptomic profile of monocytes purified from the “definite SSc” (35) and “non-fibrotic SSc” cohorts of patients and matched healthy donors (Tables 1, 2). The patient cohorts included individuals presenting with different SSc phenotypes according to clinical features and the extent of skin fibrosis, i.e., early SSc (eaSSc, *n* = 11), non-cutaneous SSc (ncSSc, *n* = 17), limited cutaneous SSc (lcSSc, *n* = 11), diffuse cutaneous SSc (dcSSc, *n* = 7).

**Figure 3:**
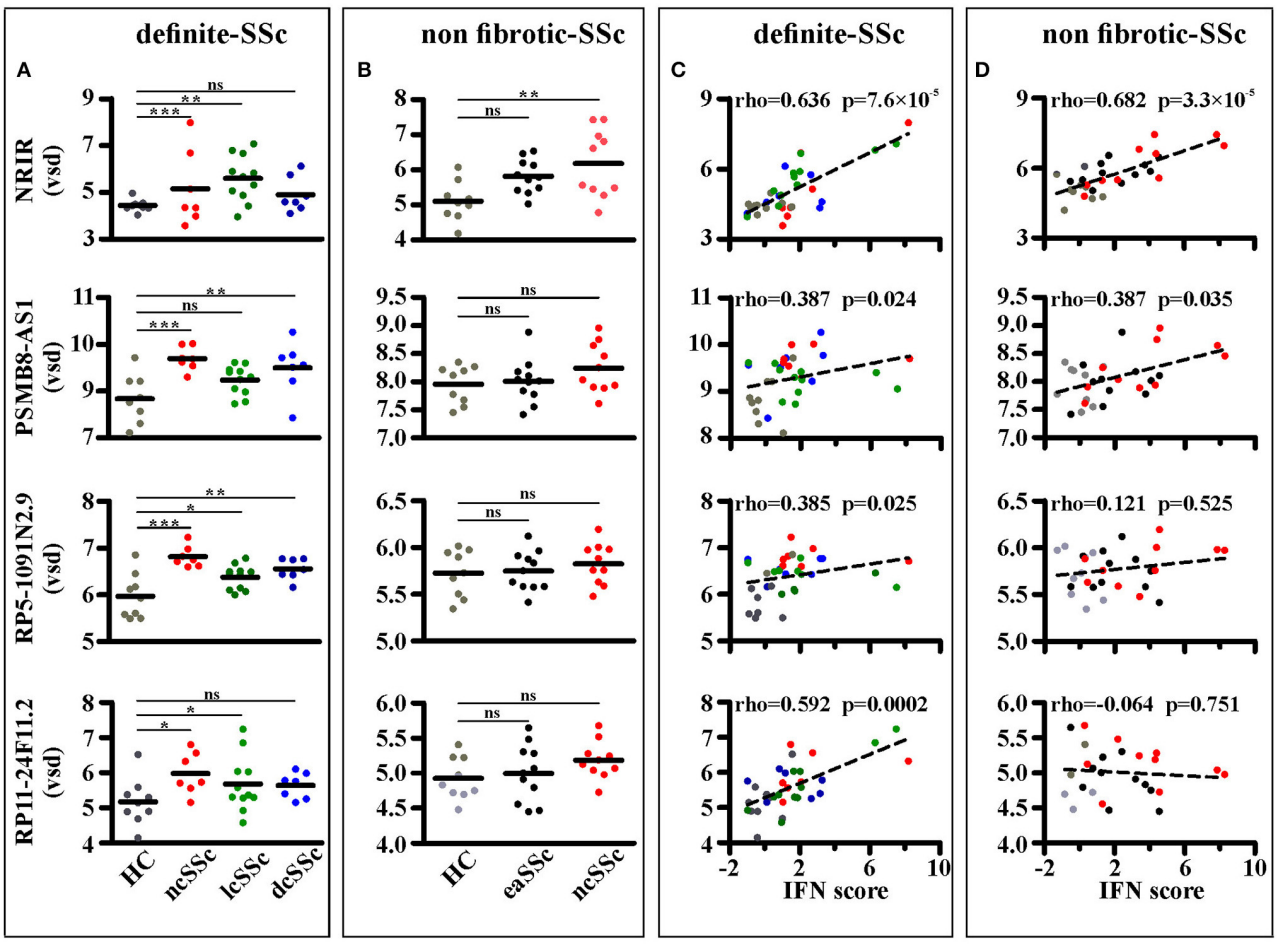
NRIR expression is increased in monocytes from SSc patients and correlates with the IFN-score. RNA sequencing data of CD14+ monocytes from SSc patients and matched healthy controls (HC) from both the definite SSc and non-fibrotic SSc cohorts were analyzed as described in Meterials and Methods. NRIR, PSMB8-AS1, RP5-1091N2.9, and RP11-24F11.2 expression were considered. LncRNAs expression in HC and patients with established Systemic Sclerosis (ncSSc, lcSSc, and dcSSc, definite-SSc cohort) **(A)** and in patients with early stages of SSc (eaSSc and ncSSc, non-fibrotic SSc cohort) **(B)** is shown. ^*^p < 0.05, ^**^p < 0.01, ^***^p < 0.001, ns, not significant, by Wald test **(C)** Correlation of NRIR expression with the IFN-score of HC (gray), ncSSc (red), lcSSc (green), and dcSSc (blue) patients is depicted. **(D)** Correlation of NRIR expression with the IFN-score of HC (gray), eaSSc (black) and ncSSc (red) patients is shown. Spearman's Rho and *p*-value are reported. NRIR expression levels are expressed as vsd, IFN Score was calculated according to Brkic et al. (16).

Four out of ninety-nine lncRNAs, namely NRIR, PSMB8-AS1, RP5-1091N2.9, and RP11-24F11.2, were expressed at significantly higher levels in at least two groups of SSc patients as compared to their respective healthy donors in the “definite SSc” cohort (Figure 3A), whereas only NRIR was significantly up-regulated in ncSSc and showed a trend in eaSSc (FC = 1.30, *p* = 0.104) in the “non-fibrotic” cohort (Figure 3B). Remarkably, only the expression of NRIR significantly correlated in both cohorts with the patients' IFN score (Figures 3C,D), calculated on the basis of the expression of IFI27, IFI44L, IFIT1, IFIT2, IFIT3, and SERPING1 (16).

IFNα was demonstrated to be central to the pathogenesis also of other systemic autoimmune diseases, with Systemic lupus erythematosus (SLE) being the prototype one. To verify whether NRIR is effectively related to the IFN signature in an *in vivo* setting in IFN-related diseases other than SSc, we retrieved from the Gene Expression Omnibus database RNA-seq data from PBMCs of SLE patients and matched healthy controls (GSE122459). Seventeen out of ninety-nine lncRNAs were commonly modulated in LPS-treated CD14+ transcriptome and SLE PBMCs compared to healthy controls (Figure S1A), and only three lncRNAs, namely NRIR, PSMB8-AS1 and RP5-1091N2.9, were modulated in all the three datasets (i.e., LPS-treated CD14+ monocytes, SSc CD14+ monocytes and PBMC from SLE patients) (Figure S1B). Remarkably, NRIR was the only one lncRNA significantly up-regulated in all the three datasets and the lncRNA most differentially expressed (log_2_FC = 1.90, *p* = 3.83 × 10^−8^) in PBMC from SLE patients as compared to healthy controls (Figure S1B).

Collectively, data from three different biological datasets (i.e., transcriptome of monocyte activated *in vitro* by LPS, transcriptome of circulating monocytes from SSc patients and transcriptome of PBMC from SLE patients) converged in identifying NRIR as belonging to the IFN signature. Therefore, we focused our study on the pathways underlying NRIR upregulation as well as on the role of this lncRNA in the type I IFN signature.

### NRIR Is a Type I IFN Dependent lncRNA

**Figure 4:**
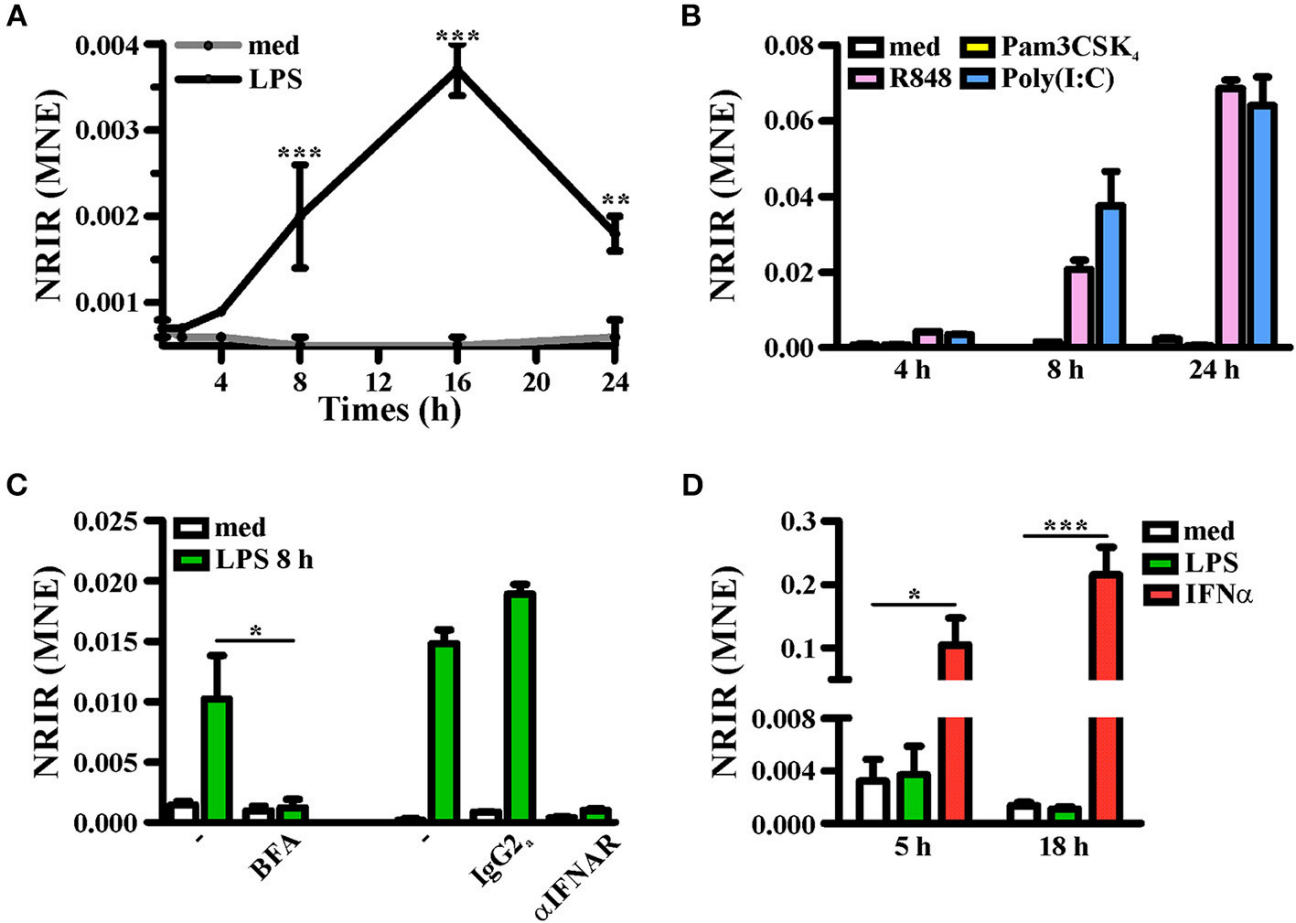
Induction of NRIR expression is IFN-dependent. **(A)** CD14+ monocytes were cultured for the indicated time point in presence of LPS (100 ng/ml, black line) or left untreated (gray line). NRIR expression levels were analyzed by RT-qPCR and expressed as mean normalized expression (MNE). Results are shown as mean ± SEM of three experiments. ^**^p < 0.01, ^***^p < 0.001 by two-way ANOVA. **(B)** CD14+ monocytes were stimulated with Pam3CSK_4_ (100 ng/ml), poly(I:C) (50 μg/ml), R848 (5 μM) or left untreated for the indicated time points. NRIR expression levels were analyzed by RT-qPCR and expressed as MNE. One experiment representative of two performed is shown. **(C)** CD14+ monocytes were stimulated with LPS or left untreated for 8 h in presence or absence of brefeldin A (BFA, left) or αIFNAR or the control IgG2a antibody (right). NRIR expression levels were analyzed by RT-qPCR and expressed as MNE. For BFA experiments results are shown as mean ± SEM of three experiments, ^*^p < 0.05 by two-way ANOVA, while for αIFNAR experiments one experiment representative of two performed is shown. **(D)** Human neutrophils were stimulated with LPS (100 ng/ml), IFNα (1,000 U/ml) or left untreated for 5 and 18 h. NRIR expression levels were analyzed by RT-qPCR and expressed as MNE. Results are shown as mean ± SEM of three experiments. ^*^p < 0.05, ^***^p < 0.001 by two-way ANOVA.

Consistent with KMC analysis of RNA-seq data that classified NRIR as a “late” transcript (Figure 1F, red line), kinetic analysis confirmed that NRIR expression is slowly induced by LPS stimulation in monocytes, being detectable after 4 h and steadily increasing over 16 h (Figure 4A). In addition, monocyte activation with agonists of TLR3 [polyinosinic:polycytidylic acid, poly(I:C)] and TLR7/8 (Resiquimod, R848), both known to promote type I IFN production, resulted in up-regulation of NRIR (Figure 4B). Conversely, a synthetic lipoprotein agonist of TLR2 (Pam3CSK4), unable to induce type I IFN transcription and secretion (47), was ineffective (Figure 4B). Consistent with this observation, treatment of monocytes with brefeldin A or with IFNα receptor (αIFNAR) blocking antibodies before LPS stimulation completely abolished NRIR induction by LPS (Figure 4C), indicating that endogenously produced type I IFNs is responsible for the upregulation of NRIR. Additionally, NRIR expression is significantly induced by IFNα but not by LPS, in human polymorphonuclear neutrophils (PMNs), that do not activate the IFN pathway downstream TLR4 (Figure 4D) (48). Taken together, these data demonstrate that type I IFN production is necessary and sufficient to increase NRIR expression in response to LPS.

### The Type I IFN-Dependent NRIR Plays a Role in the Expression of Several ISGs

Identification of pathways likely associated to NRIR function was conducted by weighted gene co-expression analysis (WGCNA). Two specific co-expression networks were created, one composed of 13 modules in the transcriptome of LPS-treated monocytes and the second one composed of 26 modules in the “definite SSc” cohort. The NRIR-related module was identified in both LPS-treated monocytes (blue module) and SSc monocytes (cyan module) co-expression networks. The blue module contained 2060 PCGs and 548 ncRNAs (Figure S2), while the cyan module was composed of 116 PCGs and 8 ncRNAs (Figure S3).

**Figure 5:**
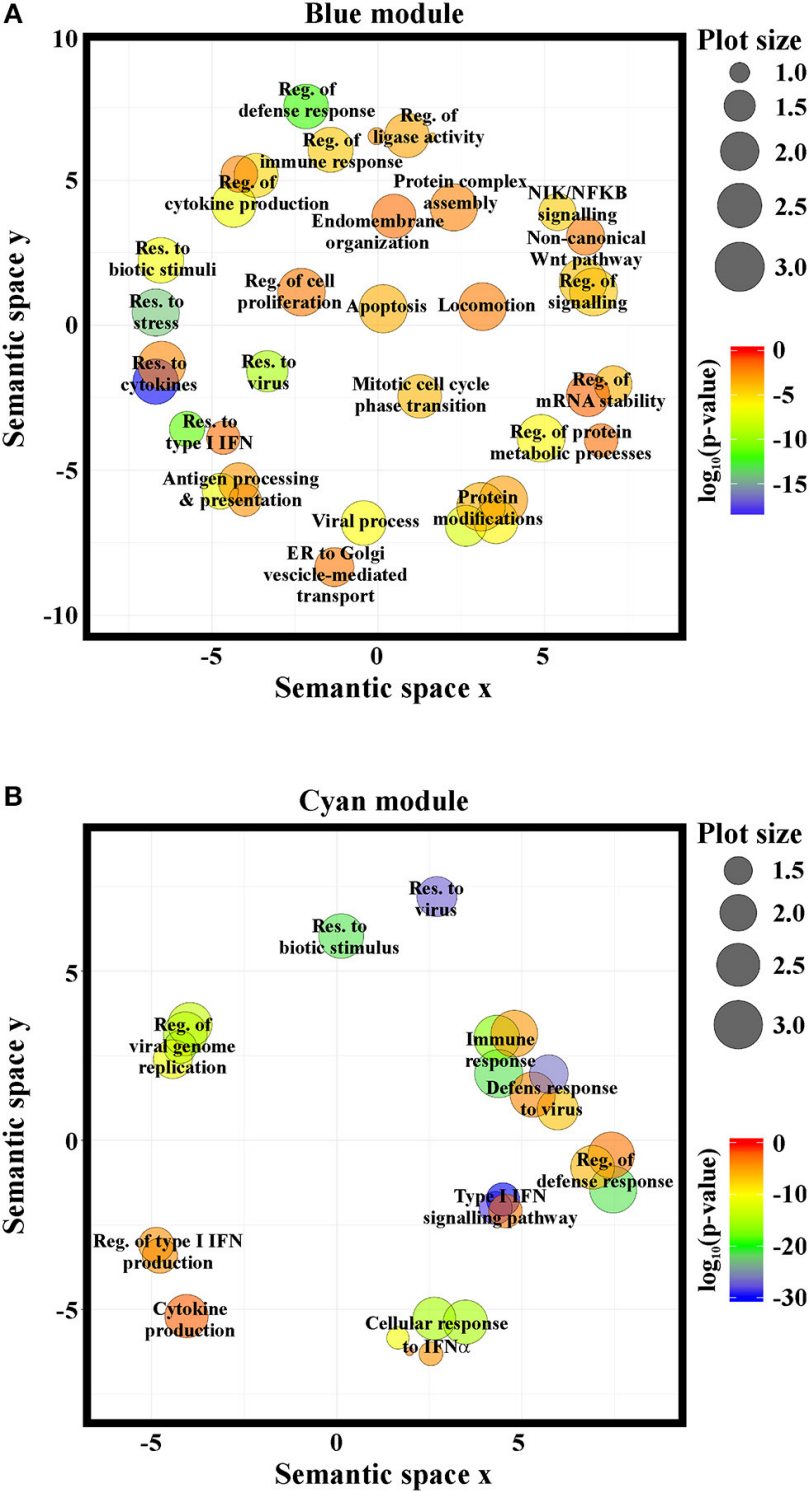
NRIR is implicated in biological processes related to immune response and the IFN/antiviral response. GO-term enrichment analysis was performed to identify biological processes enriched in the blue- **(A)** or the cyan-module **(B)**. Significantly enriched GO terms are represented as circles according to their semantic similarities. Circle size represents term specificity (bigger, general terms; smaller, specific terms), while circle color represents the log_10_ (*p*-value FDR B&H) of the enrichment.

**Figure 6:**
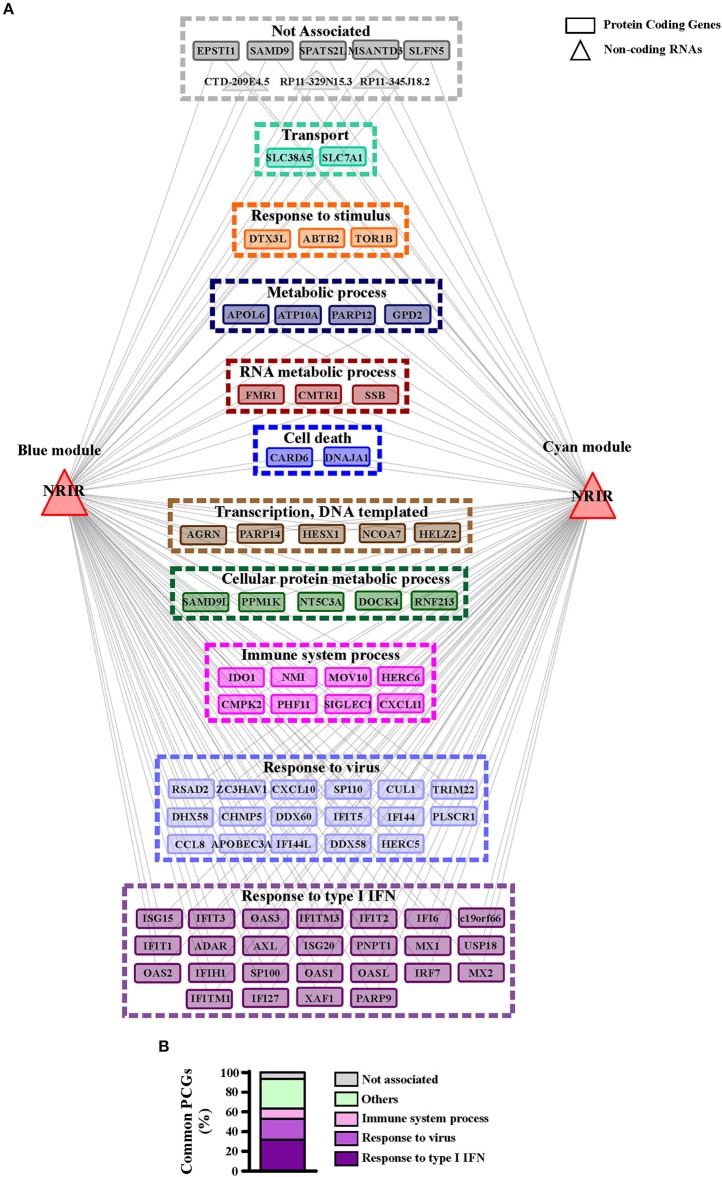
PCGs common to the blue and cyan modules are mainly involved in the immune and IFN/antiviral response. **(A)** Representation of the transcripts common to blue and cyan-module. The seventy-nine protein coding genes and the four ncRNAs are represented as rectangles and triangles, respectively. Transcripts are grouped according to their associated biological process related GO-terms. Different colors highlight different group of GO-terms, the most general GO term, summarizing each group, is reported. Genes associated to any GO-term are signed as not associated and depicted in gray. **(B)** Protein coding genes found in both modules are associated to their GO terms. Percent of common protein coding genes associated to different GO terms is shown.

GO-term and pathway enrichment analysis of the PCGs of each module underlined that biological processes related to “response to type I IFN,” “response to virus,” and “immune system process” (Figure 5) and related pathways (Tables S3, S4) were significantly enriched in both modules. Comparative analysis of the two modules identified 83 common transcripts: specifically, 79 PCGs and 4 ncRNAs (Figure 6A), the majority (63.3%) of which were associated to IFN, antiviral and immune response (Figure 6B). The 79 common PCGs were selected as putative NRIR target genes.

**Figure 7:**
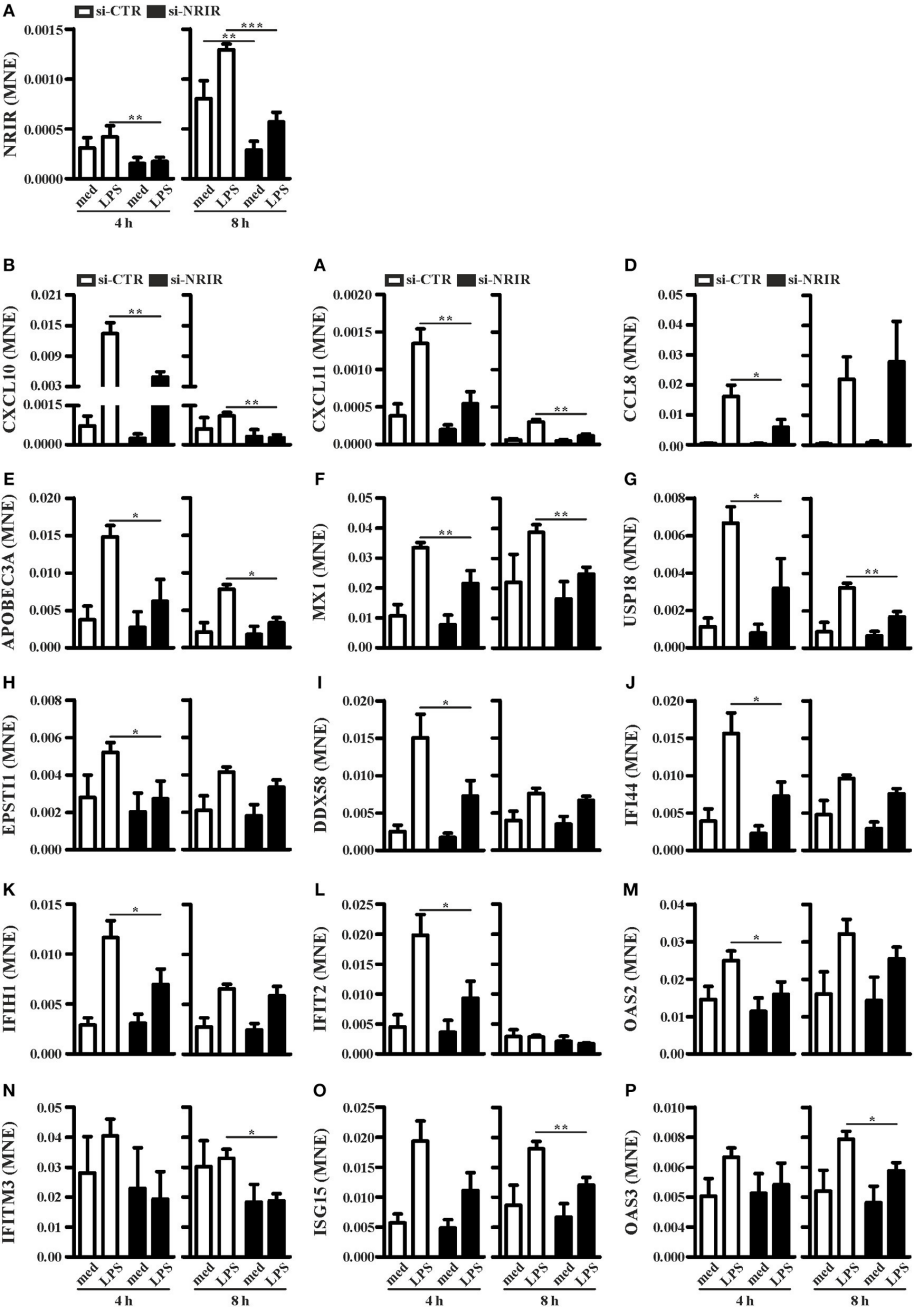
NRIR regulates fifteen of its co-expressed genes. CD14+ monocytes were transfected with si-NRIR or si-CTR and 18 h later were stimulated with LPS for 4 or 8 h or left untreated. The expression of NRIR **(A)** and its co-expressed genes **(B–P)** was analyzed by RT-qPCR and expressed as MNE. Results are shown as mean ± SEM of at least three different experiments. ^*^p < 0.05, ^**^p < 0.01, ^***^p < 0.001 by two-way ANOVA.

To investigate the role of NRIR in the regulation of IFN and anti-viral response secondary to TLR4 activation, we analyzed the expression of 56 PCGs, that were co-expressed with NRIR and common to the both blue and cyan modules (Figure 6), in NRIR-silenced monocytes. Monocyte transfection with NRIR siRNA led to an average reduction of 60.83 ± 4.81 and 55.47 ± 4.83% of the constitutive and LPS induced NRIR expression, respectively (Figure 7A). Under these conditions, the induction of fifteen PCGs by LPS was significantly impaired as compared to cells transfected with a scramble siRNA (Figures 7B–P). Precisely, decreased induction of CXCL10, CXCL11, APOBEC3A, MX1, USP18 mRNA was observed 4 h after LPS stimulation and remained reduced at 8 h as well; decreased induction of CCL8, EPSTI1, DDX58, IFI44, IFIH1, IFIT2, and OAS2 was observed at shorter time point (4 h); whereas the ability of LPS to upregulate the expression of IFITM3, ISG15 and OAS3 could be detected only at later time point (8 h) (Figures 7B–P). The induction of the remaining forty-one PCGs was unaffected by NRIR knock-down (Figures S4, S5), Strikingly, all genes modulated by NRIR silencing were also significantly upregulated in at least one group of SSc monocytes as compared to cells isolated from healthy donors (Figure S3).

**Figure 8:**
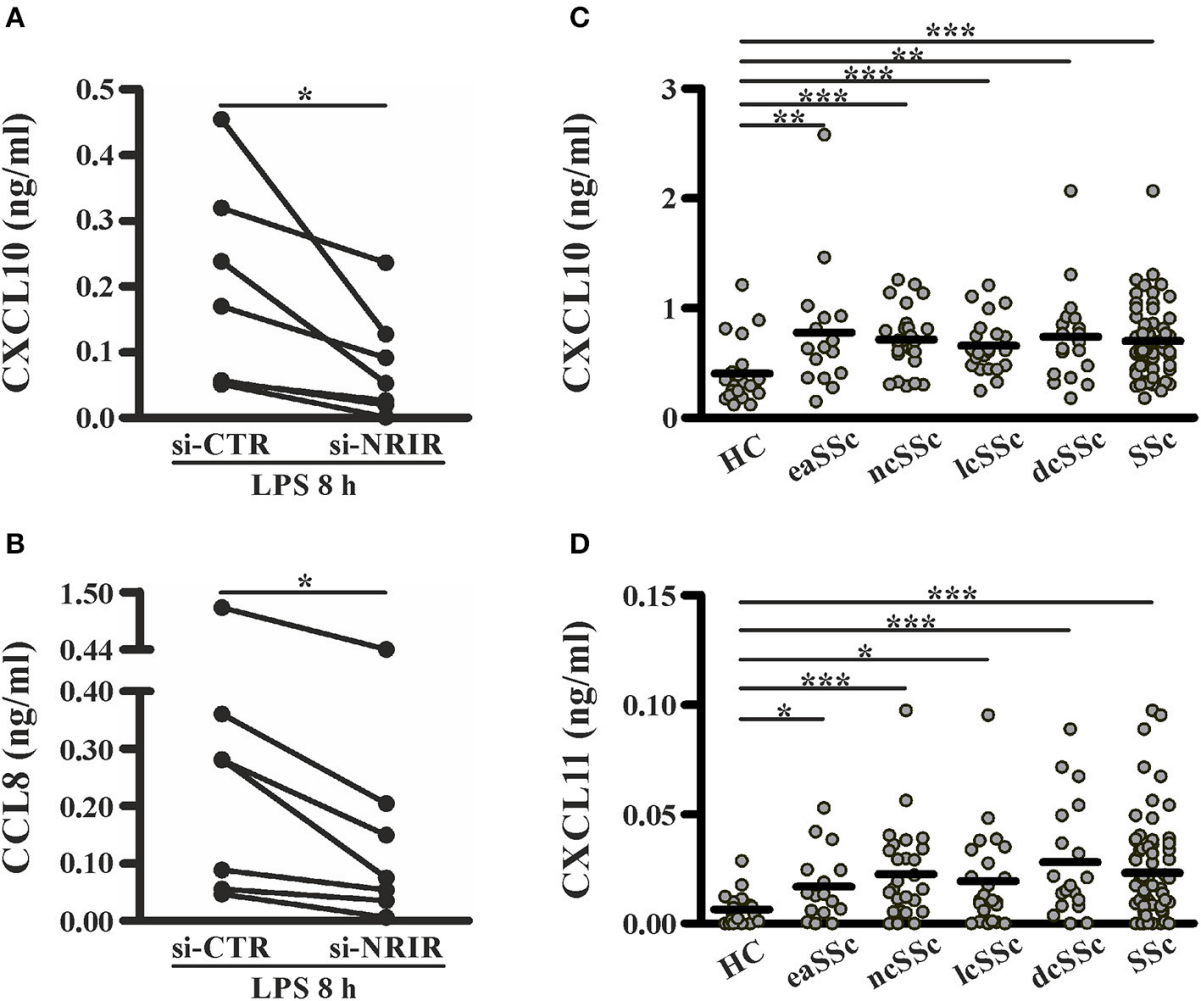
NRIR regulated proteins CXCL10 and CXCL11 are elevated in plasma of SSc patients. CD14+ monocytes from seven different donors were transfected with si-NRIR or si-CTR and 18 h later were stimulated with LPS for 8 h. Cell-free supernatants were collected, and the release of CXCL10 **(A)** and CCL8 **(B)** was measured by the multiplex immunoassay. ^*^p < 0.05 by Wilcoxon matched-pairs signed rank test. CXCL10 **(C)** and CXCL11 **(D)** level in plasma from SSc patients and matched HC was measured by the multiplex immunoassay. eaSSc, early SSc; ncSSc, non-cutaneous SSc; lcSSc, limited-cutaneous SSc; dcSSc, diffuse-cutaneous SSc. ^*^p < 0.05, ^**^p < 0.01, ^***^p < 0.001 by Mann Whitney test.

Among the IFN-responsive genes, CXCL10, CXCL11 and CCL8 have been shown to be implicated in SSc pathogenesis and/or to correlate with the degree of skin fibrosis (18, 49–51). Analysis of CXCL10, CXCL11 and CCL8 protein level in cell-free supernatants of LPS-stimulated monocytes showed a significant reduction of CXCL10 (mean reduction: 62.48 ± 8.94%, *n* = 7) and CCL8 (mean reduction: 56.13 ± 7.37%, *n* = 7) production in response to LPS (Figures 8A,B), while CXCL11 was below the detection levels (not shown). Noticeably, plasma level of CXCL10 and CXCL11 in the SSc subjects enrolled in this study was significantly higher as compared to their healthy counterparts (Figures 8C,D).

Collectively, these data substantiate the role of NRIR in the expression of several interferon-responsive genes upregulated by LPS *in vitro* or constitutively increased in circulating monocytes from SSc patients.

## Discussion

The aim of this study was to investigate the potential role of lncRNAs in the type I IFN pathway elicited in human monocytes by TLR4 activation and to explore their functional role *in vivo*, in the IFN signature displayed by SSc monocytes. Several studies have shown that lncRNAs are involved in numerous aspects of the innate and adaptive immune responses (22), and, more recently, a critical role for a small group of lncRNAs in the regulation of the IFN response has been reported (19). Likewise, evidence clearly supports the involvement of lncRNAs in the pathogenesis of autoimmune and inflammatory diseases (25, 31), where the physiologic response of immune cells is dysregulated. However, no lncRNA has been associated to the immune dysregulation present in SSc yet. Characterization of the role of lncRNAs in the regulation of monocytes IFN response to TLR4 activating agents is an important aspect to understand both the physiologic response and the disease biology of SSc arising from alteration of physiologic pathways. In fact, the link between monocytes, TLR4 activation and the downstream IFN response with SSc pathogenesis is supported by several observations: (i) circulating monocytes have been indicated as one prominent leukocyte subset playing a role in the pathogenesis of SSc (52–55); (ii) circulating SSc monocytes are characterized by an increased type I IFN signature (11, 12, 16) (iii) TLR activation may represent the connection between immune activation in SSc and tissue fibrosis (7, 10, 52, 56).

The lncRNA landscape of LPS-activated human monocytes, characterized by RNA sequencing, identified 1,278 annotated lncRNAs as upregulated and 534 as downregulated. Modulated lncRNAs were further clustered according to their kinetic of expression into early, early and transient and late. Correlation with the expression of PCGs enriched in the IFN- and anti-viral response related GO-terms allowed us to retrieve lncRNAs likely comprised into the type I IFN pathway. Moreover, as some lncRNAs have been described to regulate the expression of neighboring genes (46), lncRNAs that may have functional relevance in the expression of LPS-induced mRNAs related to the IFN/anti- viral response were retrieved on the basis of their localization *in cis* to their respective correlated PCGs.

To validate the relevance of these “IFN/viral” lncRNAs in an *in vivo* setting where the IFN response constitute a major hallmark, we examined the expression level of each of the 99 lncRNAs in monocytes from two distinct cohorts of SSc patients as compared to the relative healthy control groups. The cohorts comprised patients with the full spectrum of SSc phenotypes, from pre-clinical eaSSc, to definite groups either presenting with (lcSSc and dcSSc) or without (ncSSc) skin fibrosis. Most importantly in both cohorts a remarkable IFN signature had been identified in previous studies (16, 35). Remarkably, monocytes from lcSSc and ncSSc patients showed consistently higher levels of NRIR expression, that correlated significantly with the IFN signature in both cohorts analyzed, strikingly confirming the implication of NRIR in the IFN response also in a pathological condition. Consistently, it must be noted that NRIR had the highest expression levels in patients with ncSSc, that is the SSc subset presenting with the strongest IFN-signature (16). In addition, it is intriguing to observe that NRIR shows a trend of upregulation also in the eaSSc group, characterized by higher levels of ISGs as well. Considering that most patients with eaSSc are prompt to progress toward definite SSc (57, 58), one could speculate a potential implication of NRIR in the IFN signature intertwined with SSc progression. Remarkably, NRIR was the lncRNA most differentially expressed in PBMC from SLE patients as compared to healthy controls, thus further supporting that dysregulation of the IFN-dependent NRIR lncRNA represents a hallmark of different IFN-driven pathologies.

Identification of NRIR-related pathways was conducted according to the “guilt-by-association” method (59), that remains the only approach allowing to characterize lncRNAs based on the function of their co-expressed PCGs. NRIR was found in two distinct co-expression modules, retrieved from WGCNA analysis of the transcriptome of monocyte activated *in vitro* by LPS or isolated from SSc patients. The majority (63%) of the PCGs common to both modules was included in “response to type I IFN,” “response to virus,” and “immune system process” biological processes, thus strengthening the likelihood that NRIR plays a role in these processes. Experimental validation of the *in silico* analysis demonstrated that NRIR is a type I IFN-responsive gene, induced in monocytes upon activation of only those TLRs that can trigger type I IFN production (i.e., TLR4, TLR3 and TLR7/8). This is further supported by the demonstration that inhibition of LPS-induced release of soluble mediators, and specifically blockade of type I IFN receptor abolished the ability of LPS to upregulate NRIR. Moreover, monocyte activation with agonists of TLR2 (unable to induce type I IFN transcription and secretion) or neutrophil activation of TLR4 (that does not mobilize the TRIF-IFN pathway) (48) failed to upregulate NRIR expression.

Consistently with the NRIR role suggested by the WGCNA approach, data shows that NRIR-silencing mainly reduces the LPS-induced expression of type I IFN target genes, including, among the others, CXCL10, MX1, IFITM3, and ISG15. Moreover, measurements of CXCL10 and CCL8 secretion further endorsed the role of NRIR as a positive regulator of a subset of LPS-induced IFN-dependent genes.

The inhibition of ISGs upon NRIR-silencing is in sharp contrast with recent reports showing that NRIR acts as a negative regulator of specific ISGs (CMPK2, CXCL10, IFIT3, IFITM1, ISG15, Viperin, and IFITM3) in hepatocytes (34) or epithelial cells (60). Overall, our findings strengthen the role of NRIR as a regulator of the IFN response, but they strongly point out that NRIR function is highly cell-type or stimulus specific. Such behavior is not uncommon among lncRNAs implicated in the regulation of immune response; one example is IL7-AS, that was described either as a positive regulator of IL-6 expression in IL-1β-activate epithelial cells (61) or as negative regulator in LPS-stimulated monocytes/macrophages as well as in IL-1β activated chondrocytes (62).

It must be underlined that all the ISGs inhibited by NRIR silencing are also upregulated in SSc monocytes, that display concomitantly a prominent IFN signature as well as NRIR upregulation. These observations strengthen the relevance of the NRIR-ISGs axis in both physiological as well as pathological conditions. Among the ISGs inhibited upon NRIR silencing, numerous genes have been frequently linked to SSc. Increased levels of CXCL10, CXCL11, IFI44, and MX1 correlate with the severity of different clinical features in SSc patients (63, 64). Higher MX1 expression was associated with ischemic ulcers and reduced forced vital capacity (64, 65). The extent of skin fibrosis measured by the modified Rodman Skin Score (mRSS) correlates with the expression of IFI44 (63). Most importantly, increased levels of circulating CXCL10 and CXCL11, both NRIR targets, highly correlate with the type I IFN signature as well as with a more severe clinical phenotype, with lung and kidney involvement (11, 63, 66). In fact, serum level of CXCL10 and CXCL11 has been recently proposed as biomarker for the identification of early and non-fibrotic subset of SSc (18). Conversely, inhibition of type I IFN signature in SSc patients with anifrolumab, that blocks IFN receptor signaling, leads to lower levels of CXCL10 expression and fibrosis-related transcripts (67).

Collectively, herein we demonstrate that the IFN-dependent lncRNA NRIR is a positive regulator of the LPS-induced IFN response in human monocytes and highlight, for the first time, that aberrant expression of NRIR can be involved in the dysregulation of immune system intertwined with SSc development.

## Ethics Statement

All samples were obtained after patients provided written informed consent and after approval of the study by the institutional review board at each participating center.

## Author Contributions

BM performed experiments and analysis and contributed to write the paper, NS performed experiments and analysis on SSc monocytes, MR supervised research on SSc monocytes and wrote the paper, NT performed RNA-seq of LPS-treated monocytes, MAC supervised and critically discussed the results of the RNA-seq experiments in LPS-treated monocytes, MC, LB, and MvdK collected patients and clinical info, TR supervised the study and FB designed research and wrote the paper.

### Conflict of Interest Statement

The authors declare that the research was conducted in the absence of any commercial or financial relationships that could be construed as a potential conflict of interest.

## Footnotes

^1^https://toppgene.cchmc.org/enrichment.jsp

^2^http://www.genelink.com/tools/gl-downloads.asp

^3^http://LinRegPCR.nl

^4^http://www.gene-quantification.de/download.html.
