# Supplementary material for: The Long Non-coding RNA NRIR Drives IFN-Response in Monocytes: Implication for Systemic Sclerosis
Source: Front Immunol. 2019 Jan 31;10:100. doi: 10.3389/fimmu.2019.00100 (PMC6371048; doi:10.3389/fimmu.2019.00100)
Supplement: Supplementary file 1 [file Data_Sheet_1.docx]

Supplementary Material

The long-non-coding RNA NRIR drives IFN-response in monocytes: implication for Systemic Sclerosis

Barbara Mariotti1, Nila H. Servaas2,3, Marzia Rossato2,3,4*, Nicola Tamassia1, Marco A. Cassatella1, Marta Cossu2,3, Lorenzo Beretta5, Maarten van der Kroef2,3, Timothy R.D.J. Radstake2,3§ and Flavia Bazzoni1§

^1^Department of Medicine, General Pathology section, University of Verona, Verona, Italy

^2^Department of Rheumatology & Clinical Immunology, University Medical Center Utrecht, Utrecht University, Utrecht, The Netherlands

^3^Laboratory of Translational Immunology, Department of Immunology, University Medical Center Utrecht, Utrecht University, Utrecht, The Netherlands

^4^Department of Biotechnology, University of Verona, Verona, Italy

^5^Scleroderma Unit, Referral Center for Systemic Autoimmune Diseases, Fondazione IRCCS Ca’ Granda Ospedale Maggiore Policlinico di Milano, Milan, Italy

*** Correspondence:**

Marzia Rossato

Department of Biotechnology

University of Verona

Strada Le Grazie 15, 37134

Tel: +39 045 802 7063

[marzia.rossato@univr.it](mailto:flavia.bazzoni@univr.it)

§ co-last authors contributed equally to this work

# Figure S1


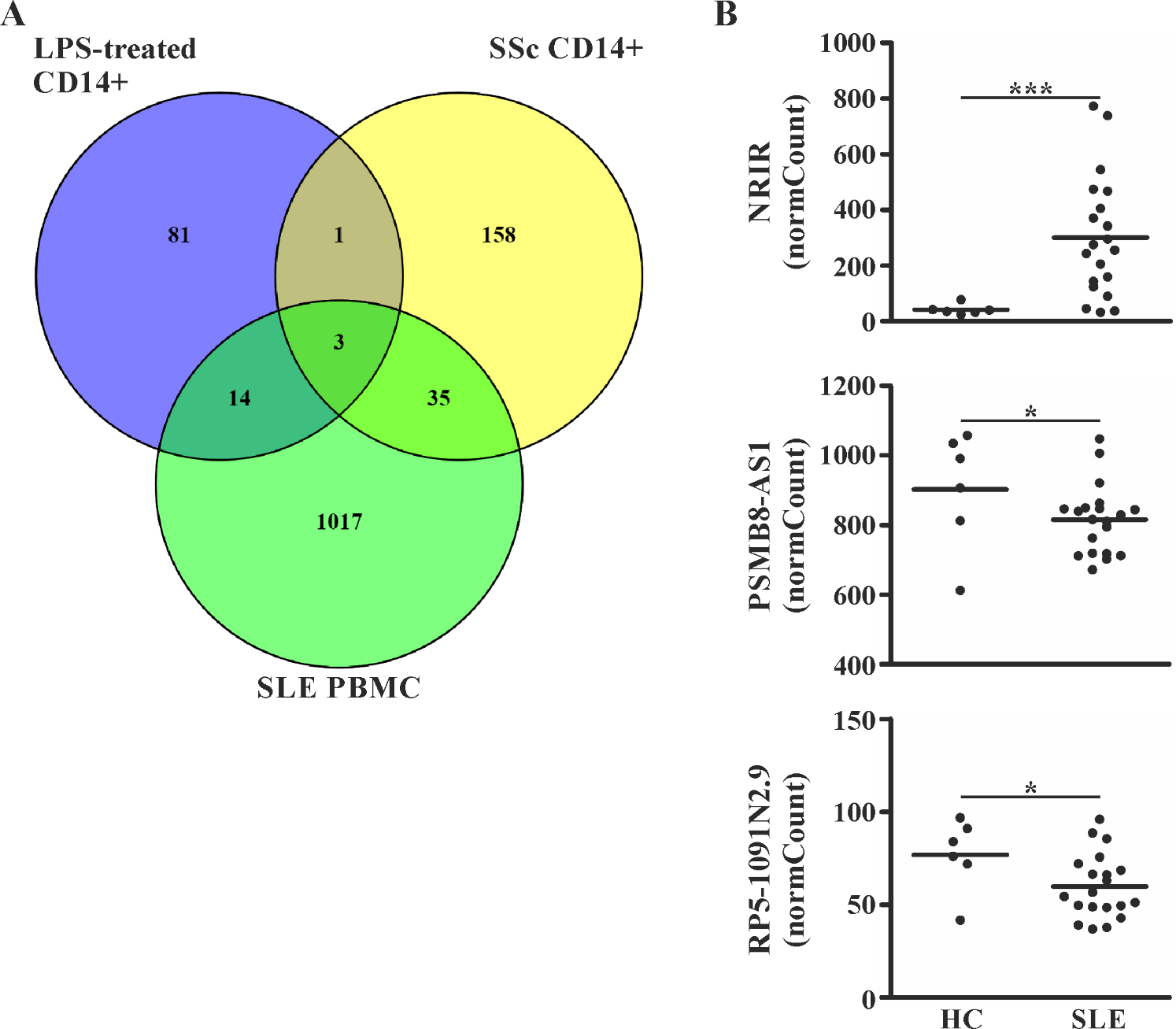


Figure S1 NRIR expression is increased in PBMC from SLE

RNA-seq data of LPS-treated CD14+ monocytes, CD14+ monocytes from SSc and matched healthy controls (HC) and PBMC from SLE patients and relative HC were analyzed as described in *Materials and Methods.* A) Venn diagram representing the IFN/viral-related lncRNAs modulated by LPS (blue), lncRNAs modulated in at least two SSc groups as compared to HC (yellow) and lncRNAs differentially expressed in PBMC from SLE patients as compared to relative HC (green). The number of specific or common lncRNAs is reported. B) Expression levels of NRIR, PSMB8-AS1 and RP5-1091N2.9 in PBMC from SLE patients as compared to HC is shown. Expression levels are reported as normalized count (normCount) according to DESeq2 normalization. * p < 0.05, *** p < 0.001 by Wald test.

#
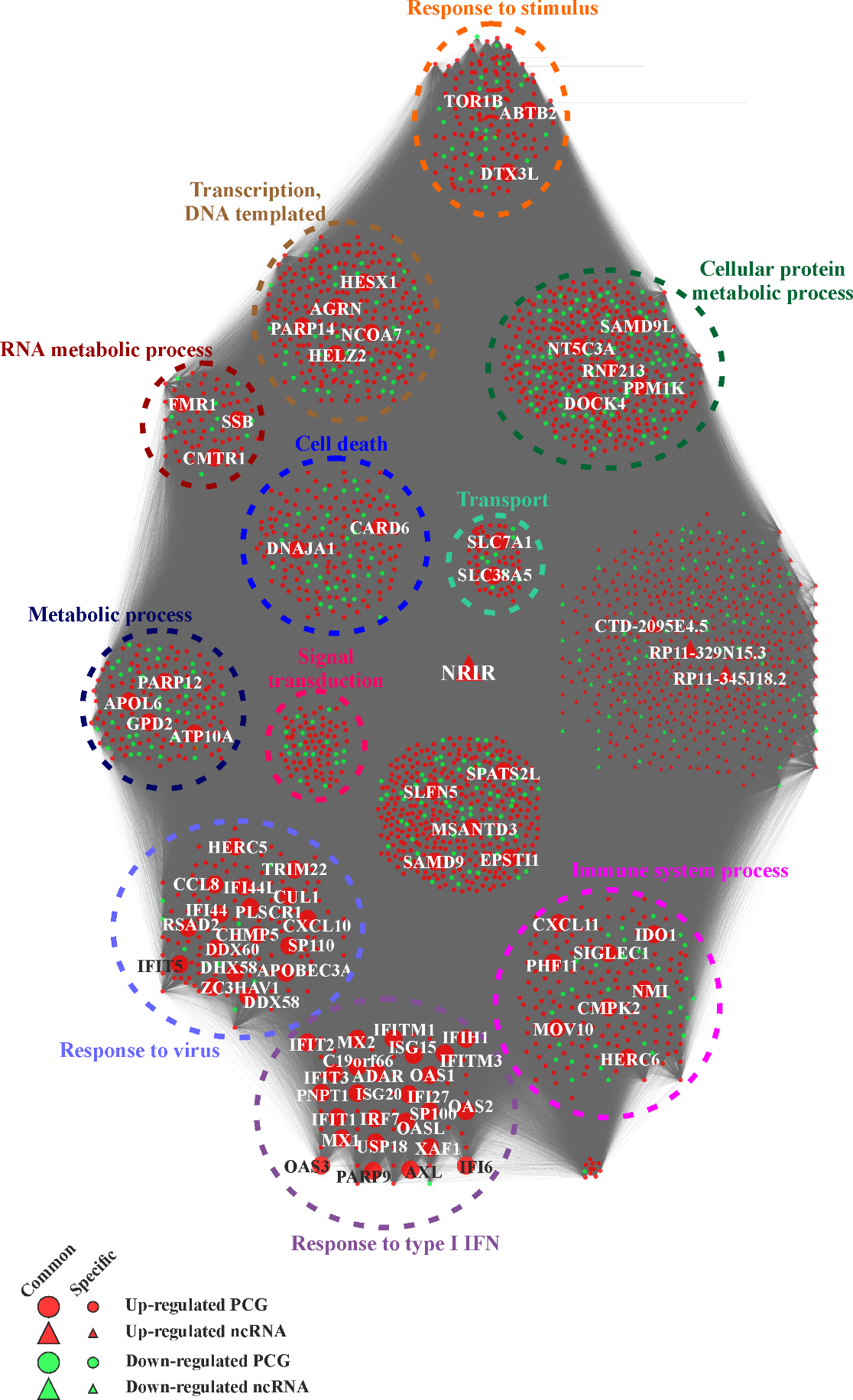
Figure S2

Figure S2 Gene co-expression network of the blue-module

Gene co-expression network was built starting from the LPS-treated and untreated monocytes transcriptome as described in *Materials and Methods.* The NRIR-associated module (blue-module) is shown. The 2060 protein coding and the 548 non-coding transcripts are represented as circles and triangles, respectively. Red nodes represent LPS-upregulated transcripts while green nodes represent down-regulated transcripts. Nodes’ size indicates the overlap between blue-module and the NRIR-associated module identified in the SSc network, where the small, unlabeled nodes represent transcripts specific of the blue-module. Transcripts are grouped according to their associated biological process related GO-terms; the most general GO term, summarizing each group is reported.

# Figure S3


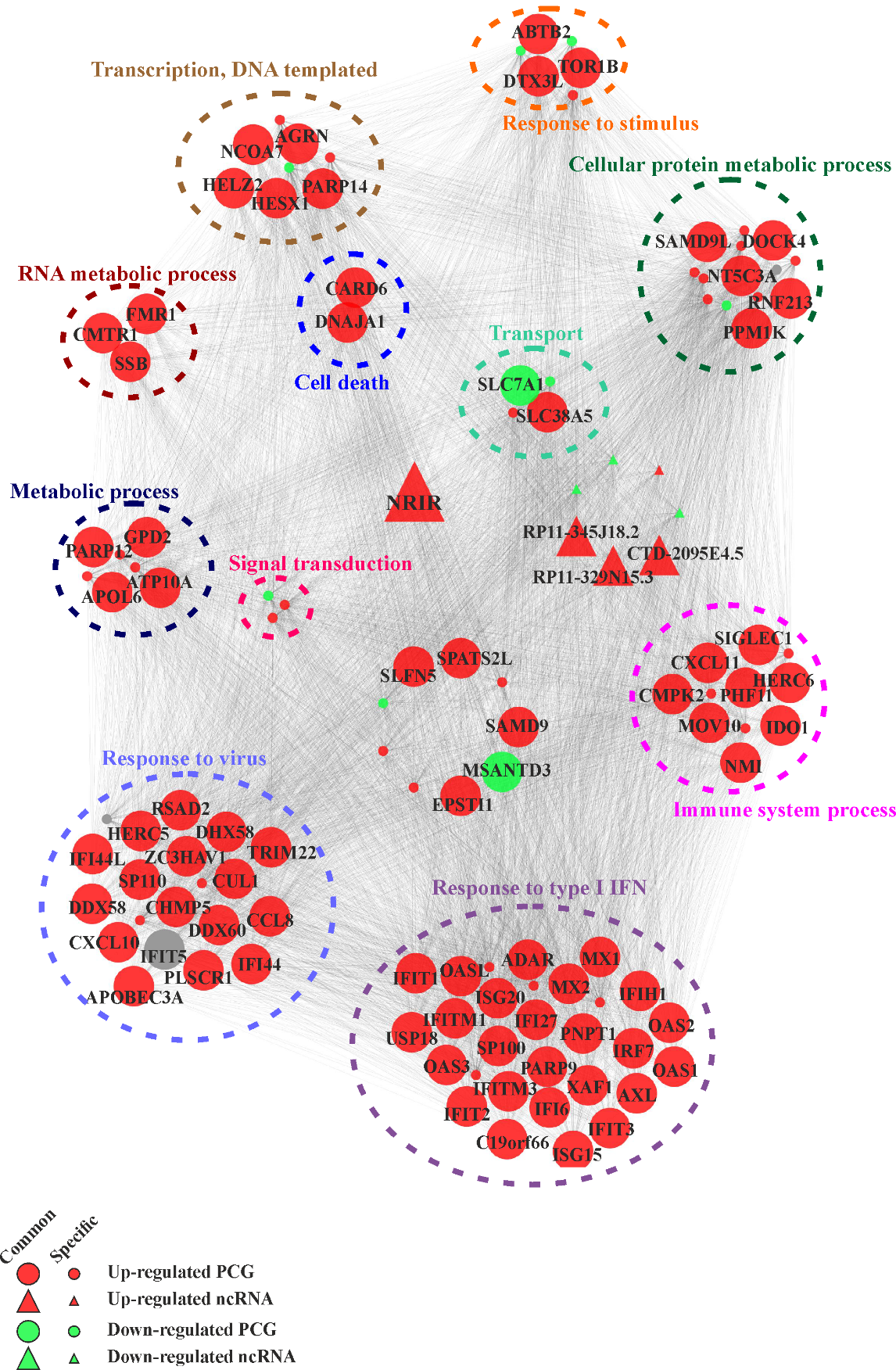


Figure S3 Gene co-expression network of the cyan-module

Gene co-expression network was built starting from the SSc monocytes transcriptome as described in *Materials and Methods.* The NRIR-associated module (cyan-module) is shown. The 116 protein coding and the 8 non-coding transcripts are represented as circles and triangles, respectively. Red nodes represent transcripts more expressed in at least one SSc subset compared to HC while, green nodes represent less expressed transcripts. Nodes’ size indicates the overlap between cyan-module and the NRIR-associated module identified in the LPS-treated monocytes network, where the small, unlabeled nodes represent transcripts specific of the cyan-module. Transcripts are grouped according to their associated biological process related GO-terms; the most general GO term, summarizing each group is reported.

# Figure S4

#
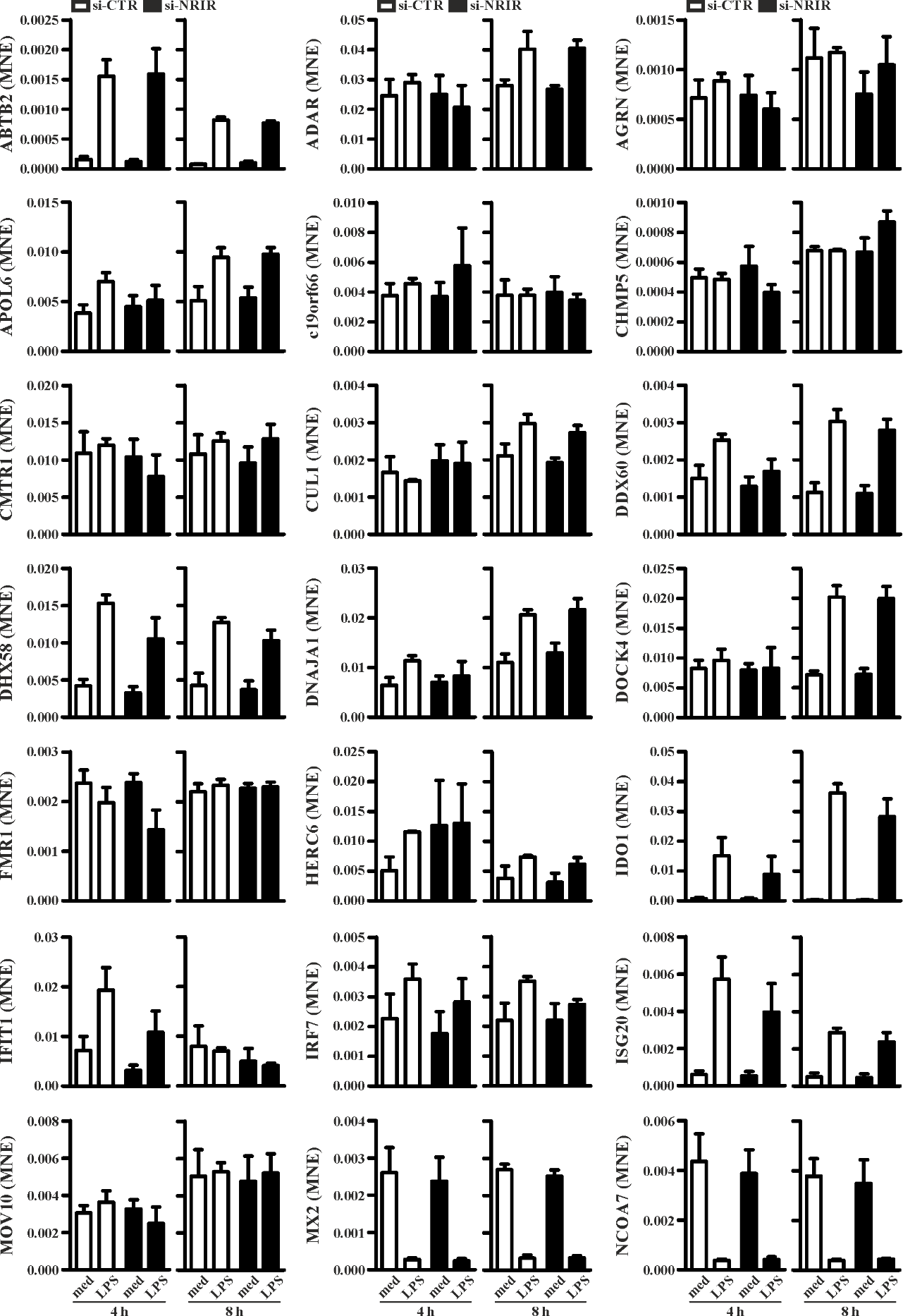


Figure S4 Effect of NRIR silencing on the expression of its putative target genes.

CD14+ monocytes were transfected with si-NRIR or si-CTR and 18h later were stimulated with LPS for 4h or 8h or left untreated. The expression of putative NRIR-target genes was analyzed by RT-qPCR and expressed as MNE. Results are shown as mean ± SEM of at least three different experiments.

# Figure S5

#
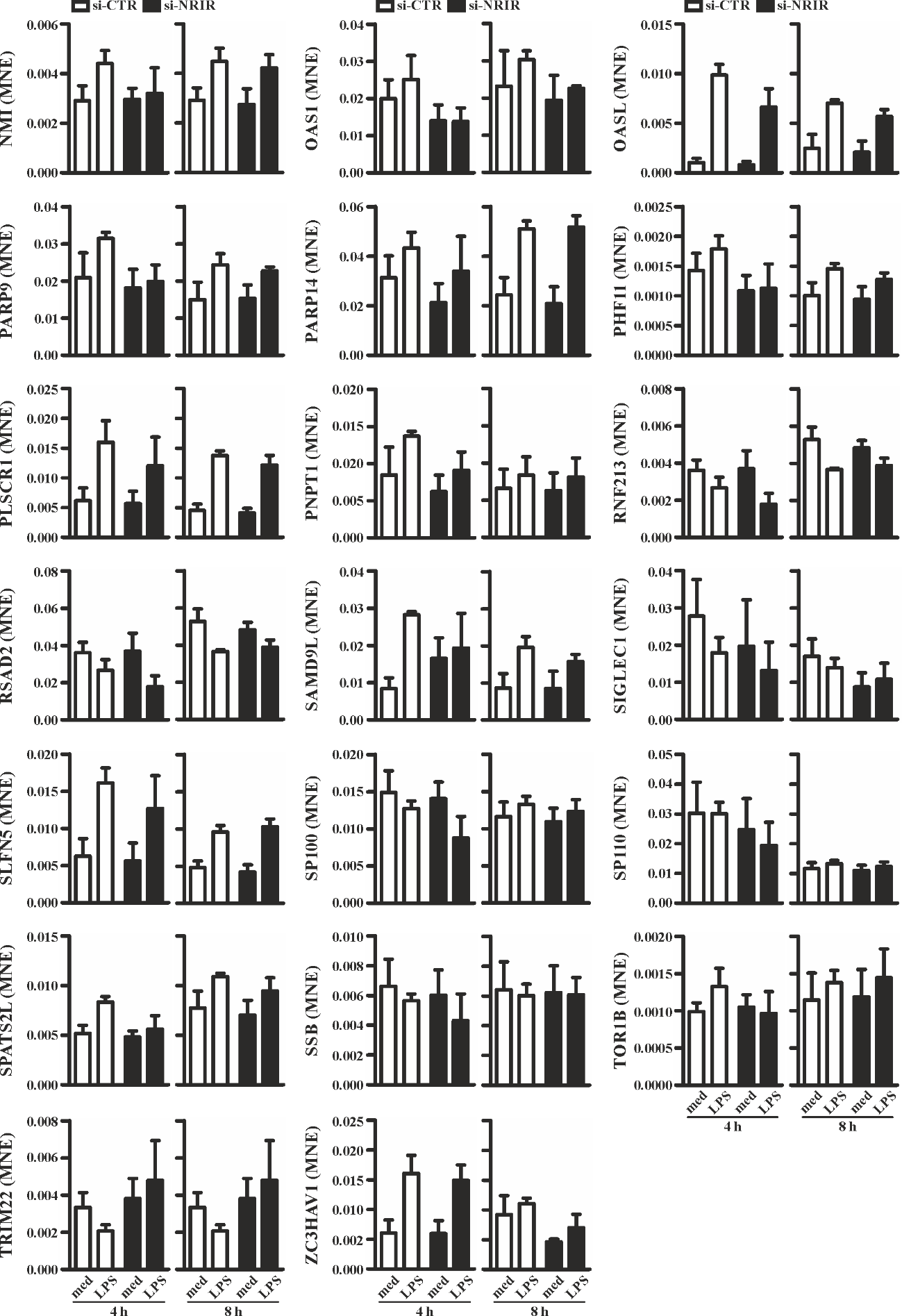


Figure S5 Effect of NRIR silencing on the expression of its putative target genes.

CD14+ monocytes were transfected with si-NRIR or si-CTR and 18h later were stimulated with LPS for 4h or 8h or left untreated. The expression of putative NRIR-target genes was analyzed by RT-qPCR and expressed as MNE. Results are shown as mean ± SEM of at least three different experiments.

# Table S1 Oligonucleotides sequences used in this study.

| siRNA sequence | | |
| --- | --- | --- |
| Targeted transcript | **Sequence** | |
| NRIR | CGATGCATGGGAAGACTAA | |
| RT-qPCR Primers | | |
|  | **Sequence** | |
| RT-qPCR Target | **Forward** | **Reverse** |
| NRIR | CTGTCTCATCCAGTGAAGAC | TTGCAGTGAGCCAATATCGC |
| ABTB2 | GACGGACCTCATCAACCAAG | CCCAGCATCAATCAACATCTG |
| ADAR | GCTCGTGAGATACCTGAACAC | CTGCTTGCCTTGCTTCTTGC |
| AGRN | GACGGAGTCACCTACGAAAAC | GCCAGCAATCACTGTCATACG |
| APOBEC3A | GGGTCACTTGGTTCATCTC | CTTGGCTGTGCTCATCTAG |
| APOL6 | GCCACCCTACTCTTCCTATC | CACCAGCCTCACTTTCTCTC |
| C19orf66 | AAGGCGGGGGCTCTGATG | GCGTAGGTTGGCTTCTGTC |
| CCL8 | GAAACCTTCACCTCTCATGCT | GAATGGAAACTGAATCTGGCTG |
| CHMP5 | GTGACTCTGCTTCCGTTTCTG | CTGCTCATACATCCTCTTTTGC |
| CMTR1 | AACGATGAAGAGGAGAACTG | CACTATCAGACCCACTAAGG |
| CUL1 | CACTGGACGACTTTAGAACATC | GGTTTGACTGGTGAACACTAG |
| CXCL10 | TTCAAGGAGTACCTCTCTCTAG | CTGGATTCAGACATCTCTTCTC |
| CXCL11 | CAAGGCTTCCCCATGTTCA | CCACTTTCACTGCTTTTACCCC |
| DDX58 | GCTCTACTAAGGGGATGATGG | CACTAAGATTCTGGCATTCTGG |
| DDX60 | TCTGGTTGAACGCTATCTTGTG | ACGCATACTCGGCATCCTTG |
| DHX58 | CCAAGCGGCACCTAGAGAC | CACGATCAGGGAGAAGACAG |
| DNAJA1 | GCTTACGAAGTTCTCTCTGATG | ACTCTACTGCTCCTTTCTTACC |
| DOCK4 | CAGAGCGGCAAGAGCATATC | CAGTGGCAAGGGAAAACAGC |
| EPSTI1 | GAGCATCAGCAATACAAAACCG | ATTTACCCTCCTGTGTTCAGTC |
| FMR1 | GCCTTGCTGTTGGTGGTTAG | CACATTTGCCGTAAGTCTTCTG |
| HERC6 | GATAGCCAAGTGTTTTCGTG | CACACCTAGATTCTTCAGTG |
| IDO1 | ATGAAGAAGTGGGCTTTGCTC | GACTTGTGGTCTGTGAGATG |
| IFI44 | GATAAAAGGGGTCATTGAGC | GAGTCACACAGAATAAACGG |
| IFIH1 | GGCATGGAGAATAACTCATCAG | CTCTTCATCTGAATCACTTCCC |
| IFIT1 | TCATCAGGTCAAGGATAGTCTG | GGTGTTTCACATAGGCTAGTAG |
| IFIT2 | ACTGCAACCATGAGTGAGAAC | GCCTCGTTTTGCCCTTTGAG |
| IFITM3 | GAATCACACTGTCCAAACCTTC | CTTCCTGTCCCTAGACTTCAC |
| IRF7 | TACCATCTACCTGGGCTTCG | AGGGTTCCAGCTTCACCA |
| ISG15 | ACTCATCTTTGCCAGTACAGGAG | CAGCATCTTCACCGTCAGGTC |
| ISG20 | AGGGAGAGATCACCGATTAC | TTCTGGATGCTCTTGTGTAG |
| MOV10 | ACTCGGGTCAGGTTCTTCAG | GGTGAGTGAGGGTAACAGAC |
| MX1 | CTGTAAATCTCTGCCCCTGTTAG | TCGTGTCGGAGTCTGGTAAAC |
| MX2 | TGCCACCACAAATGATGTTTCC | AGGGAGTCGATGAGGTCAATG |
| NCOA7 | GAAGAAGATGGTGGTTCAGAAG | TCAGTGCTATGGAGTTTAGGG |
| NMI | GAGTTACAAGAGGCTACCAAAG | GCTGTCATTCTCAGGAGTTTC |
| OAS1 | TCAGAAATACCCCAGCCAAATC | AACCAGGTCAGCGTCAGATC |
| OAS2 | CTCCCATCCTACCATTCAC | AGACTGTTTTCCGTCCATAG |
| OAS3 | GCGGGTGCTGAAAACTGTC | CATCTCACTGAGGATCTCTG |
| OASL | GGACCGTGGAGGAGTTTCTG | GAGCCCACCTTGACTACCTTC |
| PARP14 | ACCCCCCGAAGAACTTGAAC | TCCTTTTCCTTGCCATACCAAC |
| PARP9 | CTGGAAACTACAGTCTATGC | TATTCTGGAGGACTTCACAC |
| PHF11 | CACTCTGCCCCAAAGATGTC | CACAAGTCCTGAAGAATACAGC |
| PLSCR1 | CTCACAGATGAATGCTTCTCAC | GGCTGATTTGGGACAGGAAAG |
| PNPT1 | AGCGATGGTCCTTTCCTTC | ATTTCCTGTTGCCTAAGTCC |
| RNF213 | GCGACTCCTGCTCTTGCTTC | TGCCCACACTCCATTTCACC |
| RSAD2 | AGCATCGTGAGCAATGGAAG | GAAAGCGACTCTATAATCCC |
| SAMD9L | TGCTACACAGAGGATCAGAG | CAGAATTTGCCCGTATTGCTC |
| SIGLEC1 | CATCTGGTACTACGACTACTC | TGGGCTCCTCTGTTACTGTG |
| SLFN5 | ATTCTGCTGTGCGGTGTTTG | CGTTTCTGCTGCTCTTTCAG |
| SP100 | CACTGTTCAGCGATGTCAAC | CTCCCTCTCTTCTTCTTCAC |
| SP110 | GCCATACACAAGCCATTTCCC | CTCTGCTCTGCCATTCATAGG |
| SPATS2L | GCAGTTAGATCAGTTGTTCC | CTCTCCACCTTGTCTTTAGC |
| SSB | CAACAGGTTGAACCGTCTAAC | ATGAGTTCTGCCTTGGATTTGC |
| TOR1B | GGCTACCTGTCCTACAATGAC | TCACTTCCGTGGCTAGATGC |
| TRIM22 | GTCACCTCCTTCTCTATGTC | TGTGGGGAAACTCATTTGTGG |
| USP18 | GGTGTTCGTAATGAATGTGG | CAGAGTTTGAGGTACAGTTG |
| ZC3HAV1 | AGAGACCCTGCGATAACCTG | CTTTGTTCAGTCCAGAGAGTTC |
| ACTIN B | CATCGAGCACGGCATCGTCA | TAGCACAGCCTGGATAGCAAC |
| RPL32 | AGGGTTCGTAGAAGATTCAAGG | GGAAACATTGTGAGCGATCTC |

Table S2 Interferon/viral related lncRNAs and their correlated protein coding genes.

| **lncRNA** | | **PCG** | | | |
| --- | --- | --- | --- | --- | --- |
| **ENSG_ID** | **Gene Name** | **ENSG_ID** | **Gene Name** | **Rho** | **pval** |
| ENSG00000225963 | AC009950.2 | ENSG00000135899 | SP110 | 1.000 | 0.0000 |
| ENSG00000228763 | AC010095.5 | ENSG00000153201 | RANBP2 | 1.000 | 0.0000 |
| ENSG00000273237 | CTB-119C2.1 | ENSG00000122566 | HNRNPA2B1 | 1.000 | 0.0000 |
| ENSG00000275431 | CTB-186H2.3 | ENSG00000275718 | CCL15 | 1.000 | 0.0000 |
|  |  | ENSG00000271503 | CCL5 | 1.000 | 0.0000 |
|  |  | ENSG00000275385 | CCL18 | 0.943 | 0.0048 |
|  |  | ENSG00000274736 | CCL23 | 0.886 | 0.0188 |
| ENSG00000267197 | CTC-429L19.3 | ENSG00000105401 | CDC37 | 1.000 | 0.0000 |
| ENSG00000273004 | GS1-279B7.2 | ENSG00000116679 | IVNS1ABP | 1.000 | 0.0000 |
| ENSG00000223799 | IL10RB-AS1 | ENSG00000159110 | IFNAR2 | 1.000 | 0.0000 |
|  |  | ENSG00000159128 | IFNGR2 | 0.943 | 0.0048 |
| ENSG00000265666 | RARA-AS1 | ENSG00000131759 | RARA | 1.000 | 0.0000 |
| ENSG00000276809 | RP11-10E18.7 | ENSG00000102580 | DNAJC3 | 1.000 | 0.0000 |
| ENSG00000273443 | RP11-54O7.18 | ENSG00000187608 | ISG15 | 1.000 | 0.0000 |
|  |  | ENSG00000186827 | TNFRSF4 | 1.000 | 0.0000 |
| ENSG00000251230 | RP11-701P16.5 | ENSG00000151725 | CENPU | 1.000 | 0.0000 |
| ENSG00000269940 | RP11-73M18.7 | ENSG00000126214 | KLC1 | 1.000 | 0.0000 |
| ENSG00000269958 | RP11-73M18.8 | ENSG00000126214 | KLC1 | 1.000 | 0.0000 |
| ENSG00000173867 | RP11-97O12.7 | ENSG00000172183 | ISG20 | 1.000 | 0.0000 |
| ENSG00000257477 | LINC01154 | ENSG00000161638 | ITGA5 | 0.986 | 0.0003 |
| ENSG00000254233 | RP11-242J7.1 | ENSG00000151725 | CENPU | 0.986 | 0.0003 |
| ENSG00000244586 | WNT5A-AS1 | ENSG00000114251 | WNT5A | 0.986 | 0.0003 |
| ENSG00000280247 | AC005578.3 | ENSG00000105246 | EBI3 | 0.943 | 0.0048 |
| ENSG00000225964 | NRIR | ENSG00000134321 | RSAD2 | 0.943 | 0.0048 |
| ENSG00000228107 | AP000692.9 | ENSG00000159256 | MORC3 | 0.943 | 0.0048 |
| ENSG00000259758 | CASC7 | ENSG00000123908 | AGO2 | 0.943 | 0.0048 |
| ENSG00000254246 | CTB-120L21.1 | ENSG00000135077 | HAVCR2 | 0.943 | 0.0048 |
| ENSG00000258682 | CTD-2002H8.2 | ENSG00000100567 | PSMA3 | 0.943 | 0.0048 |
| ENSG00000248489 | CTD-2007H13.3 | ENSG00000153922 | CHD1 | 0.943 | 0.0048 |
| ENSG00000267607 | CTD-2369P2.8 | ENSG00000090339 | ICAM1 | 0.943 | 0.0048 |
| ENSG00000267874 | CTD-2527I21.9 | ENSG00000105707 | HPN | 0.943 | 0.0048 |
| ENSG00000214922 | HLA-F-AS1 | ENSG00000204642 | HLA-F | 0.943 | 0.0048 |
| ENSG00000224116 | INHBA-AS1 | ENSG00000122641 | INHBA | 0.943 | 0.0048 |
| ENSG00000233621 | LINC01137 | ENSG00000163877 | SNIP1 | 0.943 | 0.0048 |
|  |  | ENSG00000163874 | ZC3H12A | 0.943 | 0.0048 |
| ENSG00000204261 | PSMB8-AS1 | ENSG00000168394 | TAP1 | 0.943 | 0.0048 |
|  |  | ENSG00000204267 | TAP2 | 0.886 | 0.0188 |
| ENSG00000275260 | RP11-216P16.8 | ENSG00000135114 | OASL | 0.943 | 0.0048 |
| ENSG00000223552 | RP11-24F11.2 | ENSG00000160791 | CCR5 | 0.943 | 0.0048 |
| ENSG00000233379 | RP11-318G21.4 | ENSG00000102794 | IRG1 | 0.943 | 0.0048 |
| ENSG00000279320 | RP11-329N15.3 | ENSG00000188313 | PLSCR1 | 0.943 | 0.0048 |
| ENSG00000251136 | RP11-37B2.1 | ENSG00000104312 | RIPK2 | 0.943 | 0.0048 |
| ENSG00000260336 | RP11-395B7.7 | ENSG00000169871 | TRIM56 | 0.943 | 0.0048 |
| ENSG00000280138 | RP11-463O12.5 | ENSG00000139722 | VPS37B | 0.943 | 0.0048 |
| ENSG00000248161 | RP11-499E18.1 | ENSG00000109320 | NFKB1 | 0.943 | 0.0048 |
| ENSG00000272512 | RP11-54O7.17 | ENSG00000187608 | ISG15 | 0.943 | 0.0048 |
| ENSG00000258860 | RP11-561B11.3 | ENSG00000100906 | NFKBIA | 0.943 | 0.0048 |
| ENSG00000258137 | RP11-753H16.3 | ENSG00000161638 | ITGA5 | 0.943 | 0.0048 |
| ENSG00000240211 | RP11-758P17.3 | ENSG00000085514 | PILRA | 0.943 | 0.0048 |
| ENSG00000246451 | RP11-894P9.1 | ENSG00000126214 | KLC1 | 0.943 | 0.0048 |
| ENSG00000249487 | RP11-97O12.2 | ENSG00000172183 | ISG20 | 0.943 | 0.0048 |
| ENSG00000272084 | RP5-1126H10.2 | ENSG00000127481 | UBR4 | 0.943 | 0.0048 |
| ENSG00000272273 | XXbac-BPG252P9.10 | ENSG00000137312 | FLOT1 | 0.943 | 0.0048 |
| ENSG00000238033 | AC002480.2 | ENSG00000136244 | IL6 | 0.928 | 0.0077 |
| ENSG00000179428 | AC073072.5 | ENSG00000136244 | IL6 | 0.928 | 0.0077 |
| ENSG00000258987 | RP11-131H24.4 | ENSG00000100628 | ASB2 | 0.928 | 0.0077 |
| ENSG00000237605 | RP11-343H5.6 | ENSG00000136634 | IL10 | 0.928 | 0.0077 |
| ENSG00000229664 | RP11-536K7.5 | ENSG00000134460 | IL2RA | 0.928 | 0.0077 |
| ENSG00000228649 | AC005682.5 | ENSG00000136244 | IL6 | 0.886 | 0.0188 |
| ENSG00000235419 | AC010149.4 | ENSG00000067066 | SP100 | 0.886 | 0.0188 |
| ENSG00000266469 | CTB-131K11.1 | ENSG00000125686 | MED1 | 0.886 | 0.0188 |
| ENSG00000214894 | LINC00243 | ENSG00000137312 | FLOT1 | 0.886 | 0.0188 |
| ENSG00000267391 | MIR3591 | ENSG00000049759 | NEDD4L | 0.886 | 0.0188 |
| ENSG00000226648 | PLCG1-AS1 | ENSG00000198900 | TOP1 | 0.886 | 0.0188 |
| ENSG00000255080 | RP11-1082L8.3 | ENSG00000170873 | MTSS1 | 0.886 | 0.0188 |
| ENSG00000204277 | RP11-219G17.4 | ENSG00000184557 | SOCS3 | 0.886 | 0.0188 |
| ENSG00000227512 | RP11-413M3.4 | ENSG00000148400 | NOTCH1 | 0.886 | 0.0188 |
| ENSG00000272269 | RP11-500C11.3 | ENSG00000124789 | NUP153 | 0.886 | 0.0188 |
| ENSG00000279447 | RP4-737E23.6 | ENSG00000125810 | CD93 | 0.886 | 0.0188 |
| ENSG00000236841 | AC007750.5 | ENSG00000115267 | IFIH1 | 0.880 | 0.0206 |
| ENSG00000232949 | AC002480.4 | ENSG00000136244 | IL6 | 0.870 | 0.0244 |
| ENSG00000249738 | AC008697.1 | ENSG00000113302 | IL12B | 0.870 | 0.0244 |
| ENSG00000231290 | APCDD1L-AS1 | ENSG00000124164 | VAPB | 0.829 | 0.0416 |
| ENSG00000280087 | CTB-129P6.7 | ENSG00000130202 | PVRL2 | 0.829 | 0.0416 |
| ENSG00000271032 | CTD-2527I21.14 | ENSG00000105707 | HPN | 0.829 | 0.0416 |
| ENSG00000257621 | FLJ31306 | ENSG00000100567 | PSMA3 | 0.829 | 0.0416 |
| ENSG00000228203 | RNF144A-AS1 | ENSG00000134321 | RSAD2 | 0.829 | 0.0416 |
| ENSG00000225032 | RP11-228B15.4 | ENSG00000136807 | CDK9 | 0.829 | 0.0416 |
| ENSG00000256249 | RP11-324E6.6 | ENSG00000139722 | VPS37B | 0.829 | 0.0416 |
| ENSG00000273038 | RP11-479G22.8 | ENSG00000150093 | ITGB1 | 0.829 | 0.0416 |
| ENSG00000263826 | RP11-573D15.9 | ENSG00000156976 | EIF4A2 | 0.829 | 0.0416 |
| ENSG00000279296 | RP11-609D21.3 | ENSG00000132530 | XAF1 | 0.829 | 0.0416 |
| ENSG00000256325 | RP11-611O2.1 | ENSG00000135679 | MDM2 | 0.829 | 0.0416 |
| ENSG00000266970 | RP11-806H10.4 | ENSG00000184557 | SOCS3 | 0.829 | 0.0416 |
| ENSG00000232043 | RP4-530I15.9 | ENSG00000196396 | PTPN1 | 0.829 | 0.0416 |
| ENSG00000279164 | RP4-737E23.5 | ENSG00000125810 | CD93 | 0.829 | 0.0416 |
| ENSG00000264772 | SNORA67 | ENSG00000129245 | FXR2 | 0.829 | 0.0416 |
| ENSG00000229124 | VIM-AS1 | ENSG00000026025 | VIM | 0.829 | 0.0416 |
| ENSG00000272540 | XXbac-BPG252P9.9 | ENSG00000137312 | FLOT1 | 0.829 | 0.0416 |
| ENSG00000225541 | AC002480.5 | ENSG00000136244 | IL6 | 0.820 | 0.0458 |
| ENSG00000242317 | RP11-875H7.5 | ENSG00000114251 | WNT5A | 0.820 | 0.0458 |
| ENSG00000233818 | AP000695.4 | ENSG00000159256 | MORC3 | 0.812 | 0.0499 |
| ENSG00000229922 | RP11-240M16.1 | ENSG00000118503 | TNFAIP3 | 0.812 | 0.0499 |
| ENSG00000224969 | RP11-54O7.11 | ENSG00000187608 | ISG15 | 0.812 | 0.0499 |
| ENSG00000227039 | ITGB2-AS1 | ENSG00000197381 | ADARB1 | -0.829 | 0.0416 |
| ENSG00000275056 | RP11-127I20.8 | ENSG00000118900 | UBN1 | -0.829 | 0.0416 |
| ENSG00000276008 | RP11-137H15.4 | ENSG00000100804 | PSMB5 | -0.829 | 0.0416 |
| ENSG00000228427 | RP5-1091N2.9 | ENSG00000147168 | IL2RG | -0.829 | 0.0416 |
| ENSG00000275210 | AC008984.2 | ENSG00000275183 | LENG9 | -0.886 | 0.0188 |
| ENSG00000276529 | AP001505.10 | ENSG00000197381 | ADARB1 | -0.886 | 0.0188 |
| ENSG00000255422 | AP002954.4 | ENSG00000110367 | DDX6 | -0.886 | 0.0188 |
| ENSG00000267348 | CTB-179K24.3 | ENSG00000104856 | RELB | -0.886 | 0.0188 |
| ENSG00000188185 | LINC00265 | ENSG00000006451 | RALA | -0.886 | 0.0188 |
| ENSG00000268564 | AC003956.1 | ENSG00000141867 | BRD4 | -0.943 | 0.0048 |
| ENSG00000232224 | LINC00202-1 | ENSG00000136754 | ABI1 | -0.943 | 0.0048 |
| ENSG00000254288 | RP11-6I2.3 | ENSG00000154582 | TCEB1 | -0.986 | 0.0003 |

ENSG_ID, Ensembl Gene ID; lncRNA, long noncoding RNA; PCG, protein coding gene; Rho, Spearman Rank correlation coefficient.

Table S3 Pathways significantly enriched in blue-module.

| **Source** | **ID** | **Name** | **q-value FDR B&H** | **Count in Query List** |
| --- | --- | --- | --- | --- |
| REACTOME | 1269310 | Cytokine Signaling in Immune system | 8.88E-14 | 166 |
| REACTOME | 1269197 | ER-Phagosome pathway | 2.33E-10 | 37 |
| REACTOME | 1269195 | Antigen processing-Cross presentation | 5.88E-10 | 40 |
| REACTOME | 1269311 | Interferon Signaling | 8.66E-10 | 60 |
| REACTOME | 1269192 | Class I MHC mediated antigen processing & presentation | 8.66E-10 | 91 |
| REACTOME | 1269312 | Interferon alpha/beta signaling | 1.24E-09 | 31 |
| KEGG | 167325 | Protein processing in endoplasmic reticulum | 6.63E-09 | 51 |
| REACTOME | 1269725 | Regulation of mRNA stability by proteins that bind AU-rich elements | 3.74E-07 | 33 |
| REACTOME | 1269194 | Antigen Presentation: Folding, assembly and peptide loading of class I MHC | 2.50E-06 | 15 |
| REACTOME | 1269301 | FCERI mediated NF-kB activation | 3.70E-06 | 30 |
| REACTOME | 1269634 | Hedgehog ligand biogenesis | 3.80E-06 | 26 |
| REACTOME | 1269171 | Adaptive Immune System | 3.96E-06 | 147 |
| REACTOME | 1269318 | Signaling by Interleukins | 3.96E-06 | 104 |
| REACTOME | 1269304 | CLEC7A (Dectin-1) signaling | 3.96E-06 | 33 |
| REACTOME | 1269186 | Activation of NF-kappaB in B cells | 3.96E-06 | 26 |
| REACTOME | 1269331 | NIK-->noncanonical NF-kB signaling | 5.08E-06 | 24 |
| REACTOME | 1269841 | SCF-beta-TrCP mediated degradation of Emi1 | 5.44E-06 | 23 |
| REACTOME | 1269306 | Dectin-1 mediated noncanonical NF-kB signaling | 6.50E-06 | 24 |
| REACTOME | 1269329 | TNFR2 non-canonical NF-kB pathway | 6.53E-06 | 33 |
| REACTOME | 1269726 | AUF1 (hnRNP D0) binds and destabilizes mRNA | 6.76E-06 | 23 |
| REACTOME | 1268934 | Defective CFTR causes cystic fibrosis | 7.96E-06 | 24 |
| REACTOME | 1268932 | Disorders of transmembrane transporters | 8.02E-06 | 27 |
| MSigDB C2 BIOCARTA | M194 | Proteasome Complex | 8.02E-06 | 15 |
| REACTOME | 1268928 | Hh mutants that don't undergo autocatalytic processing are degraded by ERAD | 8.15E-06 | 23 |
| REACTOME | 1269838 | APC/C-mediated degradation of cell cycle proteins | 1.00E-05 | 29 |
| REACTOME | 1269837 | Regulation of mitotic cell cycle | 1.00E-05 | 29 |
| REACTOME | 1269314 | Interferon gamma signaling | 1.00E-05 | 30 |
| REACTOME | 1269605 | Degradation of DVL | 1.00E-05 | 23 |
| REACTOME | 1269852 | Autodegradation of Cdh1 by Cdh1:APC/C | 1.12E-05 | 24 |
| REACTOME | 1269904 | ABC-family proteins mediated transport | 1.12E-05 | 33 |
| REACTOME | 1268933 | ABC transporter disorders | 1.21E-05 | 25 |
| REACTOME | 1268927 | Hh mutants abrogate ligand secretion | 1.24E-05 | 23 |
| KEGG | 83040 | Proteasome | 1.24E-05 | 19 |
| REACTOME | 1269203 | Innate Immune System | 1.24E-05 | 209 |
| REACTOME | 1269745 | p53-Dependent G1 DNA Damage Response | 1.24E-05 | 24 |
| REACTOME | 1269744 | p53-Dependent G1/S DNA damage checkpoint | 1.24E-05 | 24 |
| REACTOME | 1457807 | FBXL7 down-regulates AURKA during mitotic entry and in early mitosis | 1.24E-05 | 22 |
| REACTOME | 1269746 | Stabilization of p53 | 1.24E-05 | 22 |
| REACTOME | 1269783 | CDK-mediated phosphorylation and removal of Cdc6 | 1.36E-05 | 21 |
| REACTOME | 1270174 | Regulation of ornithine decarboxylase (ODC) | 1.36E-05 | 21 |
| REACTOME | 1269770 | SCF(Skp2)-mediated degradation of p27/p21 | 1.36E-05 | 23 |
| KEGG | 377873 | Herpes simplex infection | 1.60E-05 | 46 |
| REACTOME | 1269844 | APC/C:Cdc20 mediated degradation of mitotic proteins | 1.63E-05 | 26 |
| REACTOME | 1269839 | Regulation of APC/C activators between G1/S and early anaphase | 1.63E-05 | 27 |
| REACTOME | 1269639 | Degradation of GLI1 by the proteasome | 1.63E-05 | 23 |
| REACTOME | 1269747 | Autodegradation of the E3 ubiquitin ligase COP1 | 1.63E-05 | 21 |
| REACTOME | 1269107 | Vpu mediated degradation of CD4 | 1.63E-05 | 21 |
| REACTOME | 1269797 | Mitotic G2-G2/M phases | 1.63E-05 | 46 |
| REACTOME | 1269193 | Antigen processing: Ubiquitination & Proteasome degradation | 1.63E-05 | 67 |
| REACTOME | 1269633 | Signaling by Hedgehog | 1.67E-05 | 38 |
| REACTOME | 1269612 | Asymmetric localization of PCP proteins | 1.67E-05 | 24 |
| REACTOME | 1269743 | G1/S DNA Damage Checkpoints | 1.67E-05 | 24 |
| REACTOME | 1269842 | Activation of APC/C and APC/C:Cdc20 mediated degradation of mitotic proteins | 1.86E-05 | 26 |
| REACTOME | 1270295 | Regulation of Apoptosis | 2.08E-05 | 21 |
| REACTOME | 1269849 | APC/C:Cdc20 mediated degradation of Securin | 2.15E-05 | 24 |
| REACTOME | 1269851 | APC/C:Cdh1 mediated degradation of Cdc20 and other APC/C:Cdh1 targeted proteins in late mitosis/early G1 | 2.25E-05 | 25 |
| REACTOME | 1269799 | G2/M Transition | 2.64E-05 | 45 |
| REACTOME | 1269845 | APC:Cdc20 mediated degradation of cell cycle proteins prior to satisfation of the cell cycle checkpoint | 2.88E-05 | 25 |
| REACTOME | 1270296 | Regulation of activated PAK-2p34 by proteasome mediated degradation | 3.20E-05 | 20 |
| REACTOME | 1270262 | Apoptosis | 3.31E-05 | 43 |
| REACTOME | 1269606 | Degradation of AXIN | 3.65E-05 | 21 |
| REACTOME | 1268714 | Asparagine N-linked glycosylation | 3.79E-05 | 61 |
| REACTOME | 1269185 | Downstream signaling events of B Cell Receptor (BCR) | 3.79E-05 | 47 |
| REACTOME | 1269638 | Degradation of GLI2 by the proteasome | 3.79E-05 | 22 |
| REACTOME | 1383017 | The role of GTSE1 in G2/M progression after G2 checkpoint | 3.79E-05 | 22 |
| REACTOME | 1269637 | GLI3 is processed to GLI3R by the proteasome | 3.79E-05 | 22 |
| REACTOME | 1269611 | PCP/CE pathway | 3.88E-05 | 29 |
| REACTOME | 1269792 | Ubiquitin-dependent degradation of Cyclin D1 | 3.88E-05 | 20 |
| REACTOME | 1269791 | Ubiquitin-dependent degradation of Cyclin D | 3.88E-05 | 20 |
| REACTOME | 1269778 | Cyclin A:Cdk2-associated events at S phase entry | 4.05E-05 | 24 |
| REACTOME | 1270261 | Programmed Cell Death | 4.58E-05 | 43 |
| REACTOME | 1270418 | Oxygen-dependent proline hydroxylation of Hypoxia-inducible Factor Alpha | 5.03E-05 | 23 |
| REACTOME | 1269487 | Signaling by SCF-KIT | 5.07E-05 | 69 |
| REACTOME | 1269769 | Cyclin E associated events during G1/S transition | 5.07E-05 | 24 |
| REACTOME | 1269479 | Downstream signal transduction | 6.13E-05 | 71 |
| REACTOME | 1269846 | Cdc20:Phospho-APC/C mediated degradation of Cyclin A | 6.47E-05 | 24 |
| REACTOME | 1269752 | Ubiquitin Mediated Degradation of Phosphorylated Cdc25A | 6.62E-05 | 20 |
| REACTOME | 1269751 | p53-Independent DNA Damage Response | 6.62E-05 | 20 |
| REACTOME | 1269750 | p53-Independent G1/S DNA damage checkpoint | 6.62E-05 | 20 |
| REACTOME | 1269610 | Beta-catenin independent WNT signaling | 6.78E-05 | 38 |
| REACTOME | 1269183 | Signaling by the B Cell Receptor (BCR) | 7.84E-05 | 52 |
| REACTOME | 1269478 | Signaling by PDGF | 8.64E-05 | 74 |
| REACTOME | 1269098 | Vif-mediated degradation of APOBEC3G | 8.67E-05 | 20 |
| REACTOME | 1269303 | C-type lectin receptors (CLRs) | 8.75E-05 | 37 |
| REACTOME | 1269836 | CDT1 association with the CDC6:ORC:origin complex | 9.17E-05 | 21 |
| KEGG | 585562 | Epstein-Barr virus infection | 1.27E-04 | 46 |
| KEGG | 217173 | Influenza A | 1.29E-04 | 41 |
| REACTOME | 1269640 | Hedgehog 'on' state | 1.43E-04 | 26 |
| REACTOME | 1269794 | Regulation of DNA replication | 1.53E-04 | 24 |
| REACTOME | 1269284 | DAP12 signaling | 1.53E-04 | 70 |
| REACTOME | 1269781 | Switching of origins to a post-replicative state | 1.53E-04 | 23 |
| REACTOME | 1269782 | Orc1 removal from chromatin | 1.53E-04 | 23 |
| REACTOME | 1269380 | Signaling by EGFR | 1.71E-04 | 71 |
| REACTOME | 1269283 | DAP12 interactions | 1.74E-04 | 72 |
| REACTOME | 1269480 | Signaling by VEGF | 1.79E-04 | 67 |
| KEGG | 122191 | NOD-like receptor signaling pathway | 1.85E-04 | 40 |
| REACTOME | 1457780 | Neutrophil degranulation | 1.97E-04 | 89 |
| REACTOME | 1269172 | TCR signaling | 2.01E-04 | 32 |
| REACTOME | 1269460 | NGF signalling via TRKA from the plasma membrane | 2.10E-04 | 74 |
| REACTOME | 1269796 | Removal of licensing factors from origins | 2.32E-04 | 23 |
| REACTOME | 1269833 | Assembly of the pre-replicative complex | 2.36E-04 | 22 |
| REACTOME | 1269176 | Downstream TCR signaling | 2.48E-04 | 28 |
| REACTOME | 1269483 | VEGFA-VEGFR2 Pathway | 2.83E-04 | 65 |
| REACTOME | 1269636 | Hedgehog 'off' state | 2.97E-04 | 28 |
| REACTOME | 1269298 | Fc epsilon receptor (FCERI) signaling | 3.05E-04 | 72 |
| REACTOME | 1269877 | Membrane Trafficking | 3.57E-04 | 105 |
| REACTOME | 1269199 | Cross-presentation of soluble exogenous antigens (endosomes) | 3.59E-04 | 18 |
| REACTOME | 1270416 | Regulation of Hypoxia-inducible Factor (HIF) by oxygen | 4.33E-04 | 23 |
| REACTOME | 1270415 | Cellular response to hypoxia | 4.33E-04 | 23 |
| REACTOME | 1270303 | Axon guidance | 4.74E-04 | 96 |
| REACTOME | 1269596 | Degradation of beta-catenin by the destruction complex | 4.95E-04 | 24 |
| REACTOME | 1269876 | Vesicle-mediated transport | 6.69E-04 | 110 |
| REACTOME | 1269506 | MAPK6/MAPK4 signaling | 6.74E-04 | 25 |
| REACTOME | 1269764 | Mitotic G1-G1/S phases | 8.18E-04 | 34 |
| KEGG | 83074 | Antigen processing and presentation | 8.61E-04 | 22 |
| KEGG | 658418 | Viral carcinogenesis | 8.66E-04 | 43 |
| REACTOME | 1270170 | Metabolism of polyamines | 1.08E-03 | 24 |
| REACTOME | 1269290 | Regulation of RAS by GAPs | 1.13E-03 | 21 |
| REACTOME | 1270414 | Cellular responses to stress | 1.22E-03 | 80 |
| REACTOME | 1269443 | Signalling by NGF | 1.27E-03 | 84 |
| KEGG | 99051 | Chemokine signaling pathway | 1.82E-03 | 39 |
| REACTOME | 1269485 | VEGFR2 mediated cell proliferation | 2.05E-03 | 51 |
| PantherDB | P00060 | Ubiquitin proteasome pathway | 2.08E-03 | 15 |
| REACTOME | 1270038 | Regulation of cholesterol biosynthesis by SREBP (SREBF) | 2.09E-03 | 17 |
| REACTOME | 1270424 | Attenuation phase | 2.10E-03 | 8 |
| KEGG | 213306 | Measles | 2.13E-03 | 31 |
| REACTOME | 1269831 | M/G1 Transition | 2.38E-03 | 23 |
| REACTOME | 1269832 | DNA Replication Pre-Initiation | 2.38E-03 | 23 |
| REACTOME | 1269198 | Endosomal/Vacuolar pathway | 2.74E-03 | 7 |
| REACTOME | 1269091 | Host Interactions of HIV factors | 2.76E-03 | 31 |
| REACTOME | 1268701 | Post-translational protein modification | 2.83E-03 | 157 |
| KEGG | 102279 | Endocytosis | 3.34E-03 | 50 |
| MSigDB C2 BIOCARTA | M5291 | Role of PI3K subunit p85 in regulation of Actin Organization and Cell Migration | 3.50E-03 | 8 |
| REACTOME | 1309122 | MAP3K8 (TPL2)-dependent MAPK1/3 activation | 3.50E-03 | 8 |
| REACTOME | 1269502 | MAPK1/MAPK3 signaling | 3.50E-03 | 49 |
| REACTOME | 1269768 | G1/S Transition | 4.20E-03 | 28 |
| REACTOME | 1269632 | Signaling by Leptin | 4.20E-03 | 49 |
| REACTOME | 1268725 | Transport to the Golgi and subsequent modification | 4.50E-03 | 35 |
| REACTOME | 1269468 | Signalling to p38 via RIT and RIN | 5.39E-03 | 48 |
| REACTOME | 1269501 | MAPK family signaling cascades | 5.44E-03 | 54 |
| REACTOME | 1269875 | DNA Replication | 5.52E-03 | 26 |
| REACTOME | 1427866 | RET signaling | 5.62E-03 | 51 |
| REACTOME | 1269323 | Interleukin-3, 5 and GM-CSF signaling | 6.11E-03 | 51 |
| REACTOME | 1269316 | Antiviral mechanism by IFN-stimulated genes | 6.37E-03 | 20 |
| REACTOME | 1269317 | ISG15 antiviral mechanism | 6.37E-03 | 20 |
| REACTOME | 1269285 | RAF/MAP kinase cascade | 6.54E-03 | 47 |
| REACTOME | 1269440 | SOS-mediated signalling | 6.54E-03 | 47 |
| REACTOME | 1269383 | SHC1 events in EGFR signaling | 6.54E-03 | 47 |
| REACTOME | 1269382 | GRB2 events in EGFR signaling | 6.54E-03 | 47 |
| REACTOME | 1268855 | Diseases of signal transduction | 6.61E-03 | 65 |
| PID | 138040 | IFN-gamma pathway | 6.61E-03 | 13 |
| KEGG | 83105 | Pathways in cancer | 6.83E-03 | 68 |
| PID | 138009 | Plasma membrane estrogen receptor signaling | 6.83E-03 | 10 |
| REACTOME | 1269322 | Interleukin receptor SHC signaling | 8.52E-03 | 48 |
| REACTOME | 1269321 | Interleukin-2 signaling | 8.59E-03 | 49 |
| REACTOME | 1269471 | ARMS-mediated activation | 9.07E-03 | 47 |
| REACTOME | 1269779 | Synthesis of DNA | 9.09E-03 | 24 |
| REACTOME | 1269465 | Signalling to RAS | 9.09E-03 | 48 |
| REACTOME | 1269464 | Signalling to ERKs | 9.15E-03 | 49 |
| REACTOME | 1269470 | Frs2-mediated activation | 9.68E-03 | 47 |
| MSigDB C2 BIOCARTA | M2529 | PDGF Signaling Pathway | 1.03E-02 | 11 |
| REACTOME | 1427863 | Ovarian tumor domain proteases | 1.06E-02 | 12 |
| REACTOME | 1270423 | HSF1-dependent transactivation | 1.09E-02 | 9 |
| KEGG | 83041 | Protein export | 1.09E-02 | 9 |
| REACTOME | 1269592 | Gastrin-CREB signalling pathway via PKC and MAPK | 1.09E-02 | 74 |
| KEGG | 1474302 | Fluid shear stress and atherosclerosis | 1.10E-02 | 30 |
| REACTOME | 1269469 | Prolonged ERK activation events | 1.11E-02 | 47 |
| REACTOME | 1270030 | PPARA activates gene expression | 1.23E-02 | 27 |
| REACTOME | 1270001 | Metabolism of lipids and lipoproteins | 1.23E-02 | 123 |
| MSigDB C2 BIOCARTA | M11520 | TPO Signaling Pathway | 1.51E-02 | 9 |
| REACTOME | 1269279 | Fcgamma receptor (FCGR) dependent phagocytosis | 1.55E-02 | 22 |
| KEGG | 469200 | Legionellosis | 1.69E-02 | 15 |
| KEGG | 83117 | Acute myeloid leukemia | 1.69E-02 | 15 |
| PID | 138057 | ErbB1 downstream signaling | 1.72E-02 | 23 |
| REACTOME | 1270029 | Regulation of lipid metabolism by Peroxisome proliferator-activated receptor alpha (PPARalpha) | 1.72E-02 | 27 |
| MSigDB C2 BIOCARTA | M7825 | Y branching of actin filaments | 1.72E-02 | 8 |
| REACTOME | 1268677 | Metabolism of proteins | 1.73E-02 | 225 |
| REACTOME | 1427860 | Deubiquitination | 1.74E-02 | 53 |
| PID | 137922 | IL12-mediated signaling events | 1.78E-02 | 16 |
| PID | 138018 | Downstream signaling in naive CD8+ T cells | 1.79E-02 | 14 |
| REACTOME | 1268726 | ER to Golgi Anterograde Transport | 1.81E-02 | 28 |
| REACTOME | 1269488 | Regulation of KIT signaling | 1.85E-02 | 7 |
| KEGG | 83051 | Cytokine-cytokine receptor interaction | 2.01E-02 | 48 |
| KEGG | 1510435 | Ferroptosis | 2.03E-02 | 12 |
| PID | 137988 | IL2 signaling events mediated by STAT5 | 2.08E-02 | 10 |
| REACTOME | 1383079 | Regulation of TP53 Degradation | 2.08E-02 | 11 |
| PID | 138024 | TGF-beta receptor signaling | 2.13E-02 | 14 |
| REACTOME | 1270304 | Semaphorin interactions | 2.14E-02 | 17 |
| PantherDB | P04393 | Ras Pathway | 2.14E-02 | 17 |
| REACTOME | 1269320 | Interleukin-1 signaling | 2.31E-02 | 13 |
| KEGG | 83056 | Ubiquitin mediated proteolysis | 2.44E-02 | 28 |
| PID | 138022 | Class I PI3K signaling events | 2.47E-02 | 12 |
| REACTOME | 1457794 | Signaling by MET | 2.48E-02 | 17 |
| REACTOME | 1269618 | IGF1R signaling cascade | 2.49E-02 | 52 |
| REACTOME | 1269620 | IRS-related events triggered by IGF1R | 2.49E-02 | 52 |
| PID | 137929 | IL27-mediated signaling events | 2.49E-02 | 9 |
| REACTOME | 1427862 | Ub-specific processing proteases | 2.49E-02 | 41 |
| REACTOME | 1383077 | Regulation of TP53 Expression and Degradation | 2.49E-02 | 11 |
| REACTOME | 1268722 | N-glycan trimming in the ER and Calnexin/Calreticulin cycle | 2.49E-02 | 11 |
| PID | 169350 | Signaling events mediated by TCPTP | 2.49E-02 | 11 |
| REACTOME | 1269518 | RHO GTPases Activate WASPs and WAVEs | 2.49E-02 | 11 |
| REACTOME | 1269299 | FCERI mediated MAPK activation | 2.50E-02 | 48 |
| REACTOME | 1270159 | Amino acid synthesis and interconversion (transamination) | 2.52E-02 | 10 |
| MSigDB C2 BIOCARTA | M1296 | IL-7 Signal Transduction | 2.53E-02 | 7 |
| REACTOME | 1269617 | Signaling by Type 1 Insulin-like Growth Factor 1 Receptor (IGF1R) | 2.58E-02 | 52 |
| REACTOME | 1269777 | S Phase | 2.68E-02 | 27 |
| REACTOME | 1268727 | COPII (Coat Protein 2) Mediated Vesicle Transport | 2.74E-02 | 17 |
| MSigDB C2 BIOCARTA | M18719 | Role of ERBB2 in Signal Transduction and Oncology | 2.97E-02 | 8 |
| GenMAPP | MAP00480_Glutathione_metabolism | MAP00480 Glutathione metabolism | 2.97E-02 | 8 |
| REACTOME | 1269901 | Scavenging by Class F Receptors | 2.97E-02 | 4 |
| BIOCYC | 1108771 | allopregnanolone biosynthesis | 2.97E-02 | 4 |
| PID | 138000 | IL23-mediated signaling events | 3.02E-02 | 11 |
| MSigDB C2 BIOCARTA | M1001 | Rho cell motility signaling pathway | 3.11E-02 | 10 |
| PID | 137991 | ErbB4 signaling events | 3.11E-02 | 10 |
| KEGG | 82979 | Amino sugar and nucleotide sugar metabolism | 3.11E-02 | 13 |
| KEGG | 812256 | TNF signaling pathway | 3.12E-02 | 23 |
| REACTOME | 1269903 | Transmembrane transport of small molecules | 3.16E-02 | 102 |
| PantherDB | P00006 | Apoptosis signaling pathway | 3.22E-02 | 22 |
| REACTOME | 1270312 | NCAM signaling for neurite out-growth | 3.36E-02 | 48 |
| PID | 137932 | IL6-mediated signaling events | 3.37E-02 | 12 |
| GenMAPP | MAP00970_Aminoacyl_tRNA_biosynthesis | MAP00970 Aminoacyl tRNA biosynthesis | 3.45E-02 | 7 |
| REACTOME | 1269429 | Insulin receptor signalling cascade | 3.45E-02 | 51 |
| MSigDB C2 BIOCARTA | M8615 | IL-2 Receptor Beta Chain in T cell Activation | 3.63E-02 | 11 |
| REACTOME | 1457806 | RAB GEFs exchange GTP for GDP on RABs | 3.82E-02 | 20 |
| KEGG | 114228 | Fc gamma R-mediated phagocytosis | 3.82E-02 | 20 |
| KEGG | 83077 | Jak-STAT signaling pathway | 3.83E-02 | 30 |
| PID | 138053 | TNF receptor signaling pathway | 4.03E-02 | 12 |
| REACTOME | 1269428 | Signaling by Insulin receptor | 4.22E-02 | 54 |
| KEGG | 694606 | Hepatitis B | 4.35E-02 | 28 |
| KEGG | 173973 | Hepatitis C | 4.45E-02 | 26 |
| REACTOME | 1269431 | IRS-mediated signalling | 4.45E-02 | 50 |
| KEGG | 692234 | PI3K-Akt signaling pathway | 4.54E-02 | 56 |
| PID | 138002 | E-cadherin signaling in keratinocytes | 4.67E-02 | 7 |
| KEGG | 1474301 | IL-17 signaling pathway | 4.79E-02 | 20 |
| PantherDB | P00049 | Parkinson disease | 4.94E-02 | 19 |
| GenMAPP | MAP00052_Galactose_metabolism | MAP00052 Galactose metabolism | 4.94E-02 | 8 |

B&H, Benjamini&Hochberg; FDR, False Discovery Rate; KEGG, Kyoto Encyclopaedia of Genes and Genomes; PID, Protein Interaction Database.

Table S4 Pathways significantly enriched in cyan-module.

| **Source** | **ID** | **Name** | **q-value**  **FDR B&H** | **Count in**  **Query List** |
| --- | --- | --- | --- | --- |
| REACTOME | 187104 | Interferon alpha/beta signaling | 2.35E-26 | 20 |
| REACTOME | 187103 | Interferon Signaling | 5.00E-22 | 23 |
| REACTOME | 366171 | Cytokine Signaling in Immune system | 3.06E-18 | 24 |
| KEGG | 217173 | Influenza A | 2.42E-06 | 11 |
| KEGG | 213306 | Measles | 1.87E-05 | 9 |
| KEGG | 377873 | Herpes simplex infection | 2.25E-05 | 10 |
| REACTOME | 530760 | Antiviral mechanism by IFN-stimulated genes | 2.25E-05 | 7 |
| REACTOME | 530761 | ISG15 antiviral mechanism | 2.25E-05 | 7 |
| WikiPathways | 198918 | Type II interferon signaling (IFNG) | 3.94E-05 | 6 |
| KEGG | 117292 | RIG-I-like receptor signaling pathway | 2.60E-04 | 6 |
| REACTOME | 187106 | Interferon gamma signaling | 2.60E-04 | 6 |
| REACTOME | 187097 | RIG-I/MDA5 mediated induction of IFN-alpha/beta pathways | 4.77E-04 | 6 |
| KEGG | 173973 | Hepatitis C | 8.48E-04 | 7 |
| REACTOME | 106579 | Formation of editosomes by ADAR proteins | 1.08E-03 | 2 |
| REACTOME | 106578 | mRNA Editing: A to I Conversion | 1.08E-03 | 2 |
| REACTOME | 106580 | C6 deamination of adenosine | 1.08E-03 | 2 |
| KEGG | 125137 | Cytosolic DNA-sensing pathway | 1.38E-03 | 5 |
| REACTOME | 187102 | Negative regulators of RIG-I/MDA5 signaling | 1.58E-03 | 4 |
| REACTOME | 187098 | TRAF3-dependent IRF activation pathway | 2.02E-03 | 3 |

B&H, Benjamini&Hochberg; FDR, False Discovery Rate; KEGG, Kyoto Encyclopaedia of Genes and Genomes.
